# Transcriptomic Study Reveals Widespread Spliced Leader *Trans*-Splicing, Short 5′-UTRs and Potential Complex Carbon Fixation Mechanisms in the Euglenoid Alga *Eutreptiella* sp.

**DOI:** 10.1371/journal.pone.0060826

**Published:** 2013-04-09

**Authors:** Rita C. Kuo, Huan Zhang, Yunyun Zhuang, Linda Hannick, Senjie Lin

**Affiliations:** 1 Department of Marine Sciences, University of Connecticut, Groton, Connecticut, United States of America; 2 J. Craig Venter Institute, Rockville, Maryland, United States of America; J. Craig Venter Institute, United States of America

## Abstract

*Eutreptiella* are an evolutionarily unique and ecologically important genus of microalgae, but they are poorly understood with regard to their genomic make-up and expression profiles. Through the analysis of the full-length cDNAs from a *Eutreptiella* species, we found a conserved 28-nt spliced leader sequence (Eut-SL, ACACUUUCUGAGUGUCUAUUUUUUUUCG) was *trans*-spliced to the mRNAs of *Eutreptiella* sp. Using a primer derived from Eut-SL, we constructed four cDNA libraries under contrasting physiological conditions for 454 pyrosequencing. Clustering analysis of the ∼1.9×10^6^ original reads (average length 382 bp) yielded 36,643 unique transcripts. Although only 28% of the transcripts matched documented genes, this fraction represents a functionally very diverse gene set, suggesting that SL *trans*-splicing is likely ubiquitous in this alga’s transcriptome. The mRNAs of *Eutreptiella* sp. seemed to have short 5′- untranslated regions, estimated to be 21 nucleotides on average. Among the diverse biochemical pathways represented in the transcriptome we obtained, carbonic anhydrase and genes known to function in the C_4_ pathway and heterotrophic carbon fixation were found, posing a question whether *Eutreptiella* sp. employs multifaceted strategies to acquire and fix carbon efficiently. This first large-scale transcriptomic dataset for a euglenoid uncovers many potential novel genes and overall offers a valuable genetic resource for research on euglenoid algae.

## Introduction

Euglenoid algae are a unique group of algae that are phylogenetically a sister group of Kinetoplastida, which often are parasitic [1]. Ecologically they are important primary producers in the marine coastal ecosystems. Their nutritive value (*e.g.* rich in vitamins and lipid, [2], [3]) makes them ideal prey for heterotrophic organisms. In some marine ecosystems euglenoid algae can account for a substantial fraction of the total Chlorophyll *a* [4]. The genus *Eutreptiella* (Class Euglenoidea) comprises nine known marine and brackish water species ([5] and refs therein), which can be dominant phytoplankton seasonally [6]–[10]. For instance, *E. braarudii* can make up to 30 to 46% of the total phytoplankton population in some areas [5]. Some of the *Eutreptiella* species (*e.g., Eutreptiella gymnastica*) can form blooms [5], [8], especially in nutrient-rich coastal or brackish waters [5], [8], [11], [12]. They can migrate vertically to explore resources at different depths and form cysts to survive adverse environmental conditions [8]. With the widespread and often abundant presence indicated by these previous studies [4]–[10], *Eutreptiella* and other euglenoid algae can have a profound impact on the energy flow and nutrient cycling in the coastal marine ecosystem. Transcriptomic information would help us understand the molecular mechanisms underpinning the physiological and ecological potential of this important group of microalgae. However, a broad-scale study of the transcriptomic and genetic characteristics for euglenoids have remained unexplored.

In a phylogenetically disjointed group of eukaryotes, such as trypanosomes, flatworms, hydra, rotifers, chordates and dinoflagellates, nucleus-encoded mRNAs often undergo spliced leader (SL) *trans-*splicing, which tailors the 5′ ends of the mRNAs by adding a short conserved RNA fragment (∼15–50 nt depending on species) [13]. This conserved SL is transplanted from a short non-coding RNA (SL RNA). As the SL sequence is lineage specific, it can be a useful tool for transcriptomic work on a certain lineage, especially when the organism to be studied co-exists with others organisms in a culture (*e.g.* as prey [14]) or in the natural environment (*e.g.* as sympatric organisms [15]). In the phylum Euglenozoa, SL *trans-*splicing has been reported for kinetoplastid parasites such as trypanosomes [16] and euglenoid algae such as *Euglena* [17]. Here, we detected the presence of SL *trans-*splicing in *Eutreptiella* sp. in a culture isolated from Long Island Sound [18], where this and related species contributed significantly to total phytoplankton pigment [19], [20], and even formed blooms [21]. We then investigated the distribution of the SL in the transcriptome of this alga and analyzed SL-based transcriptomes derived from cultures grown under different conditions to gain insights into the genetic potential of this alga.

## Methods

### Culture Preparation and RNA Isolation

*Eutreptiella* sp. strain LIS2002 was originally isolated from Long Island Sound, Connecticut, USA in 2002 [18]. Cultures were maintained in f/2 medium (without silicate) prepared with filtered natural seawater at 20°C and the salinity of 28 practical salinity unit (PSU) under a 12∶12 h light-dark cycle with photon fluence rate of 100 µE m^−2 ^s^−1^. Cell counts were carried out for each culture every other day. DNA was isolated from the culture periodically and used as the template for PCR amplification of the small subunit rRNA gene (18S rDNA) with a reported primer set [22]. The 18S rDNA amplicons were directly sequenced to confirm that there was no contamination of other eukaryotic algae in the cultures (GenBank accession # JQ337867). To investigate the SL sequence in this species, about 10^9^ *Eutreptiella* cells were harvested during the exponential growth phase by centrifugation at 3000×g at 15°C for 20 min. The cell pellet was resuspended thoroughly in TRIzol solution (Invitrogen) for RNA extraction.

To characterize the transcriptome and its SL prevalence in *Eutreptiella* sp., two sets of cultures were grown in phosphate-depleted and phosphate-replete f/2 medium for broadening gene diversity in the samples. For each culture, cells were also harvested in the light (1 hour before light off) and dark periods (1 hour after light off) and preserved in TRIzol as described above. These time points were expected to provide good chance to obtain genes involved in regulation of cell division and lipid production, which we were interested in. For each TRIzol-preserved sample, total RNA was isolated following Zhang et al. [23], from which mRNA was further isolated using PolyA Tract mRNA System (Promega). RNA quantities were measured using NanoDrop 1000 (Thermo Scientific), and qualities were assessed using the ratio of absorbance at 260 nm to that at 280 nm.

### Full-length cDNA Prepared Using GeneRacer Kit

About 200 ng mRNA extracted from the samples was treated with calf intestinal phosphatase (CIP) to dephosphorylate truncated mRNA, followed by tobacco acid pyrophosphate (TAP) treatment to decap the full-length mRNA, and ligation of a GeneRacer^TM^RNA Oligo to the 5′ end of the treated mRNA following the manufacturer’s protocol (Life Technologies, Grand Island, NY, USA). The treated mRNA was used to synthesize cDNA with a modified oligo dT according to Zhang et al. [23]. After purification using Zymo DNA Cleanup & Concentration column (Zymo Research, Orange, CA), PCR amplification for the full-length double-stranded cDNAs was performed with the 1^st^ strand cDNA using ExTaq (Clontech, Mountain View, CA, USA) with the primer set GeneRacer5’ (5′-CGACTGGAGCACGAGGACACTGA-3′) and GeneRacer3’ (5-GCTGTCAACGATACGCTACGTAACG-3′). PCR consisted of one cycle of 95°C for 1 min followed by 30 cycle of 95°C for 10 sec and 68°C for 4 min. The amplicons with sizes >500 bp were recovered from a 1% agarose gel using Zymoclean™ Gel DNA Recovery Kit (Zymo Research) and cloned into a T-vector. Clones were picked randomly and sequenced using Sanger technique according to Zhang et al. [23].

### 454 Sequencing of the SL-based Transcriptomes

Based on the SL sequence of *Eutreptiella* sp. (Eut-SL) found (for details see Results section), we designed primers to PCR-amplify the 5′-end of the cDNAs for 454 transcriptome sequencing. The mRNA isolated from the cultures under the four conditions was used as the template to separately synthesize cDNA with an oligo primer named 454AT_7_N_9_ (5′-CGTATCGCCTCCCTCGCGCCATCAGTAATACGACTCACTATAGGGAGNNNNNNNNN-3′, where N is any of the 4 nucleotides), which was adopted from Moreno-Paz M and Parro [24] by modifying the primer to suite 454 sequencing. The synthesized 1^st^ strand cDNA was used as the template for PCR amplification of the transcriptome using ExTaq with 454AT_7_ (5′-CGTATCGCCTCCCTCGCGCCATCAGTAATACGACTCACTATAGGGAG-3′) as the reverse primer and a modified Eut-SL primer (454BEutSL, 5′-GAGACTATGCGCCTTGCCAGCCCGCTCAGACACTTTCTGAGTGTCTATTTCTTTTCG-3′) as the forward primer. The PCR amplification was carried out for each library under a touch-down PCR program: 94°C for 1 min; 95°C for 15 sec, 60°C for 30 sec, 72°C for 2 min for 10 cycles; 95°C for 15 sec, 52°C for 30 sec, 72°C for 2 min for 10 cycles. After agarose gel electrophoresis of the amplicons, the 300–500 bp and 500–700 bp size fractions were recovered. Both of the selected fractions were subjected to emulsion PCR using GS Titanium SV emPCR Kit and then sequenced using GS Titanium Sequencing Kit (Roche) on the GS FLX System at the Center for Applied Genetics and Technology, University of Connecticut, USA.

### Sequence Processing and Clustering

Raw sequencing reads from all of the 4 libraries were pooled together and trimmed using CLC Genomics Workbench (CLC Bio, Aarhus, Demark). The alignment scores for trimming SL primer (28 nt located at 5′ end) were set up as follows: mismatch cost 1, gap cost 1, internal matches 25 that allows 3 mismatches and minimum score 24 that allows 4 mismatches at end matches. To achieve a 5′-end complete cDNA dataset, sequences without SL were discarded. Next, 50 nt from 3′ end were trimmed to remove primer-adaptor sequence (454AT_7_N_9_ oligo). Subsequently, terminal regions of the resulting sequences were checked for quality, and were removed if quality score was low (<20%) or ambiguous nucleotides occurred. Finally, after these quality and primer trimmings, sequences shorter than 150 nt were discarded.

In order to filter out 454 sequencing errors and create a redundancy-reduced sequence dataset, USEARCH [25] was used for sequence clustering. To combine identical sequences, the sequences were first sorted by length and clustered based on 100% sequence identity. Then, the consensus sequences (redundancy-reduced sequences) from the first clustering were sorted by size (the number of the sequences in a contig) and clustered again based on 97% sequence identity to remove sequence errors as USEARCH calculates the majority vote for each column (i.e. the nucleotides of the seed sequences) [25]. Web-based DeconSeq (http://edwards.sdsu.edu/cgi-bin/deconseq/deconseq.cgi) [26] was used to identify possible sequence contamination. The screening was carried out with default thresholds (query coverage ≥90% and identity ≥94%) using human and bacterial databases containing 1,116 genomes and 76,337 whole genome shotgun sequences.

Following clustering, transcripts were annotated using BLASTx algorithm [27] in Blast2Go V.2.5.0 [28] against GenBank non-redundant (nr) database, with a cut-off-E-value ≤10^−3^. Enzyme commission (EC) numbers, Gene Ontology (GO) terms associated with biological process, molecular function, and cellular component were retrieved. The GI numbers of top hits from BLASTx results were fed into Galaxy (http://usegalaxy.org/) to obtain taxonomic information by using the program of Fetch Taxonomic Representation under the Function of Metagenomic Analyses [29]–[31]. Top-hit species were binned into different phyla.

### Assessing Sequencing Depth

Post-clustering unique transcript sequences and their respective frequencies in the cDNA libraries were subjected to rarefaction curve analysis using the analytical approximation algorithm in Analytic Rarefaction V.1.3 (http://strata.uga.edu/software/index.html). Rarefaction curve analysis was originally developed to estimate sampling effort and species diversity in ecology [32] but has been adopted to evaluate sequencing depth in metagenomics of microbial communities [33], [34], next generation sequencing [35], [36] or transcriptome studies [37].

### Examination of the Length of 5′- Untranslated Regions of mRNAs

Genes with BLAST hits (E-value ≤10^−3^) were used to locate the canonical start codon AUG. Open reading frames (ORFs) were identified based on BLASTX results and the first AUG within the frame was predicted as start codon. The distance between Eut-SL and the predicted start codon (the 5′-untranslated region, 5′-UTR) was estimated using scripts written in Python 2.5.2 and Biopython (http://tiopython.org/, [38]). In an attempt to verify the results, we next selected a subset of 100 genes with significant BLAST hit (E <10^−15^) to genes with conserved start codons and manually estimated the length of 5′-UTR for each gene. We also investigated the sequences flanking the putative start codon AUG for Kozak-like sequence, the consensus sequence found in mRNAs of various eukaryotes [39], which plays a major role in the initiation of the translation process [40], and the Kozak-like sequences found in Euglenozoa [41] and land plants [42]. The consensus of BLASTx alignment (i.e. AUG occurring in the same position as the start codon AUG in hit sequence) and Kozak-like sequence prediction was used to yield a validated putative start codon dataset, and the positions of putative start codons were further inspected manually to ensure accuracy; the resultant data were then used to estimate the 5′-UTR.

## Results

### Spliced Leader of *Eutreptiella* sp. and its Application to Transcriptome 454 Sequencing

**Figure 1:**
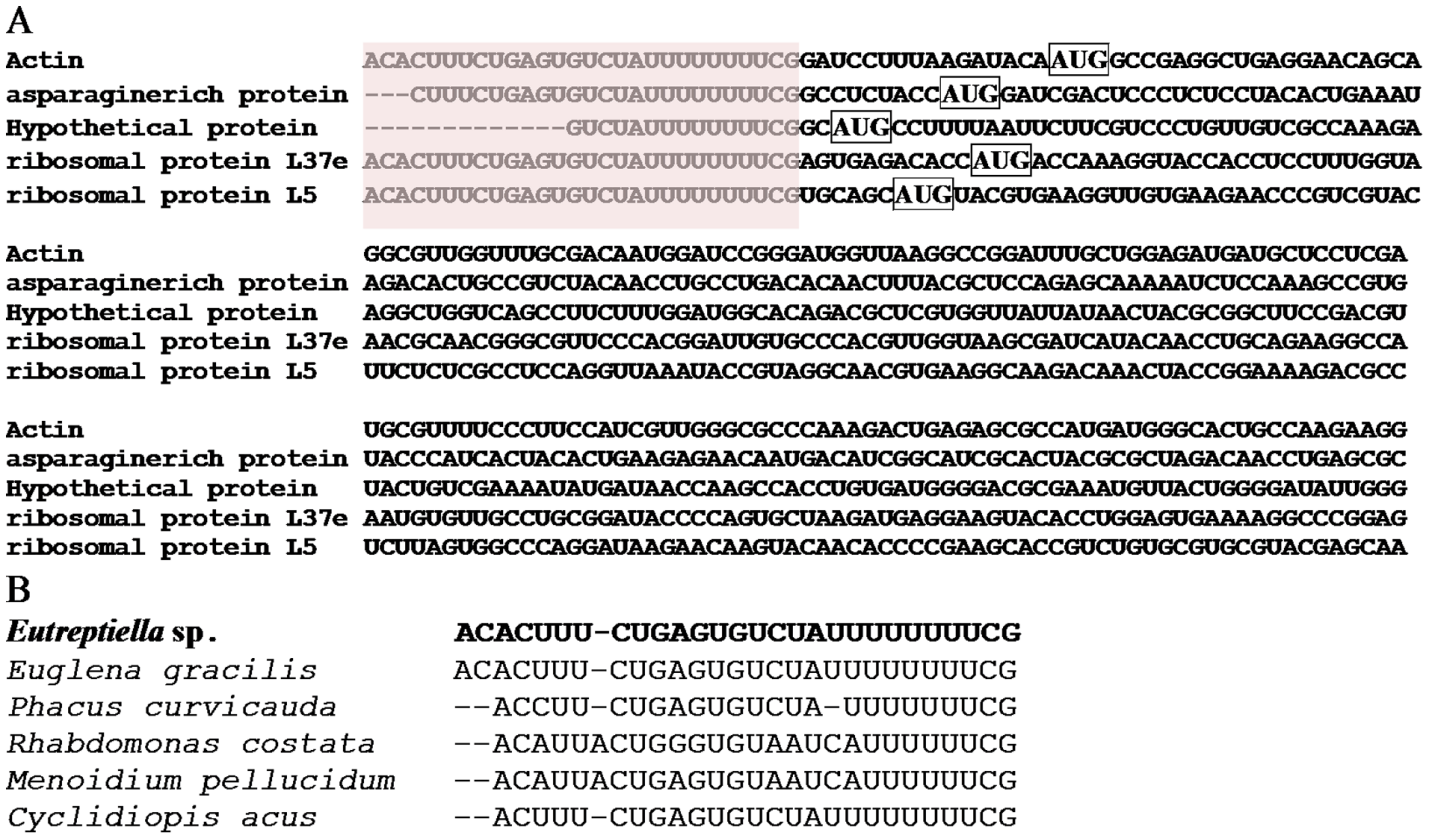
Spliced Leader (SL) trans-splicing in *Eutreptiella* sp. (A) SL sequence found in five *Eutreptiella* cDNAs. The 28-nt identical SL is shaded. The potential start codon ATG is boxed. (B.) SL in *Eutreptiella* sp. (bold-typed) and other euglenoids (reported by Frantz et al. [17]).

Three full-length cDNA clones encoding different genes were found with a 28-bp identical sequence (5′- ACACUUUCUGAGUGUCUAUUUUUUUUCG-3′) at the 5′-ends, with two more cDNAs containing partial of the sequence (Figure 1A). BLAST search against GenBank nr database hit the reported SL sequences of other euglenoid algae such as *Euglena gracilis* and *Phacus curvicauda* with 100% identity to the SL sequence in *E. gracilis* and high similarity to the SL in other Euglenozoa [17] (Figure 1B), indicating that this sequence was the SL of *Eutreptiella* sp. (named Eut-SL).

**Figure 2:**
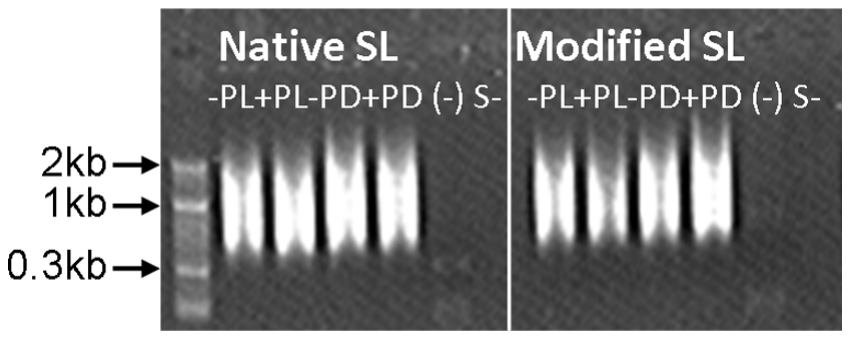
Agarose gel electrophoresis of *Eutreptiella* sp. spliced leader (Eut-SL)-based cDNA libraries. Native SL, cDNA amplified with native Eut-SL pairing with 454AT7 primer; Modified SL, cDNA amplified with modified Eut-SL pairing with 454AT7 primer. −PL: the phosphate-depleted-light sample; +PL: the phosphate-replete-light sample; −PD: the phosphate-depleted-dark sample; +PD: the phosphate-replete-dark sample. (−) negative controls without cDNA template; S-, negative controls of PCR with SL single primer.

To avoid error in reading the single nucleotide repeats in 454 sequencing, we replaced a “T” in the repetitive T tract (eight Ts) with a “C” in the Eut-SL primer, and compared the PCR efficiencies of this primer and the native Eut-SL by pairing them with 454AT7 primer separately in PCR reactions. Using the cDNA synthesized with oligo 454AT_7_N_9_ as the template, both of the primer sets were able to amplify quite evenly in the size range from 0.4 kb to >2 kb (as indicated by the even smears of cDNA; see Figure 2). Since the primer sets were designed to amplify the 5′-end region of the transcripts in random size ranges, theoretically shorter amplicons (300–500 bp) in the libraries would better represent the whole transcriptome because they could cover both the longer (>500 bp) and shorter (<500 bp) protein coding transcripts. To maximize the coverage of the transcriptome and fully exploit the read length of 454 sequencing (>400 bp), we selected 300–500 bp and 500–700 bp size fractions of the PCR amplicon to sequence for each library.

### Clustering of the Transcriptome Data

A total of 1,891,627 reads were generated in two 454 full-plate runs from samples of contrasting light and nutrient conditions. The average length was 382 bp. After quality trim and primer trim, the average length was 314 bp, with 243,551 sequences that did not meet the specified standard being discarded. The remaining 1,648,076 good-quality reads were then clustered using UClust in USEARCH [25]. A total of 1,636,130 reads were clustered into 24,697 contigs while the remaining 11,946 reads were identified as singletons (32.6% of the unique sequences), yielding 36,643 unique transcripts [contigs+singletons; Table 1. Transcript read length 150–508 bp, available upon request to the authors. The 454 raw reads were deposited to Sequence Read Archive (SRA) database of NCBI with the SPR accession numbers SRP019053 to SRP019056]. This indicated that the SL *trans*-spliced transcript pool represented a high number of unique genes. None of the 36,643 unique transcripts were flagged as contaminated sequences from bacteria or human by DeconSeq (Figure S1); direct sequencing of 18S rRNA gene PCR amplicon showed that the sequences were identical to what we previously reported for *Eutreptiella* sp. (GenBank accession number JQ337867). The results indicated that using PolyA Tract mRNA System to first isolate eukaryotic mRNA and then using *Eutreptiella* SL primer to specifically amplify *Eutreptiella* transcriptome would avoid obtaining sequences from potential contaminants (e.g. bacteria or human). Rarefaction analysis suggested that our 454 sequencing depth did not yet reach the asymptote gene number in this species, because the number of unique genes continued to increase with sequencing effort (the number of reads) within our sampling scale (Figure S2).

**Table 1:** Summary of *Eutreptiella* sp. transcriptome 454 sequencing and data analysis.

	Number of sequences	Average read length (bp)
**454 sequencing**		
Raw sequencing reads	1,891,627	382
**Trimming**		
Eliminated sequences	243,551	<150
Trimmed sequences	1,648,076	314
**Clustering**		
Reads clustered as contigs	1,636,130	336.5
Number of contigs	24,697	336.5
Average reads per contig	45	
Singletons	11,946	281.5
Unique transcripts	36,643	318.5
Percentage of singletons	32.6%	
**Annotation**		
Unknown transcripts	26,487	
Annotated transcripts	10,156	
Percentage of annotated transcripts	27.7%	
Transcripts assigned with enzyme code	1,038	
Percentage of transcripts with enzyme code	2.8%	

Transcript sequences were compared to GenBank non-redundant (nr) database using the BLASTx algorithm [27], with a cut-off E-value ≤10^−3^.

### Gene Discovery from 454-sequenced Transcriptome Dataset

Blast2Go [28] was used to annotate the sequences and obtain GO terms and EC numbers. A total of 10,156 sequences (27.7% of unique sequences) were annotated with significant BLAST results. Among those annotated sequences, 1,038 were assigned enzyme codes (2.8% of transcripts) (Table 1) and 5,330 had paralogs, which were assigned into 1,363 genes. Genes or gene families that potentially have 10 or more than 10 paralogs are listed in Table S1.

**Figure 3:**
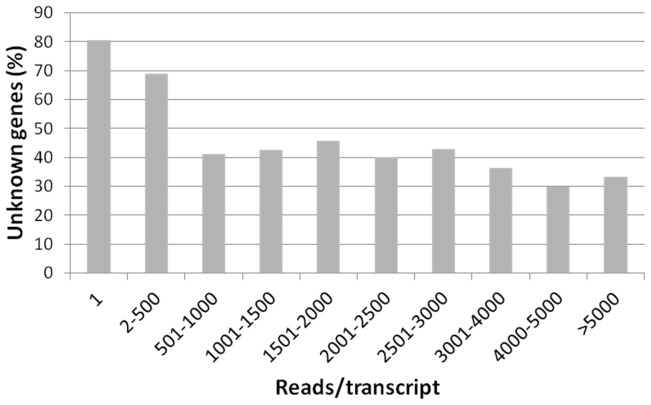
The percentage of unknown genes in *Eutreptiella* transcripts. The transcripts were assigned into different categories based on the number of reads in each transcript. In general, transcripts represented by high numbers of reads have less proportions of unknown genes.

Annotation results revealed a large proportion of unknown, potentially novel, genes. Overall, about 72% of the cDNAs did not have significant BLAST hits in the NCBI nr database. These unknown genes showed lower expression levels than those that matched documented genes (Figure 3). Reciprocally, about 80% of the singletons were unknown. In contrast, lower proportions of the unknown genes were represented by high read numbers (i.e. highly transcribed). Even so, about 37% of the transcripts that were represented by over 3000 reads had no significant BLAST hits.

### Short 5′-UTR

**Figure 4:**
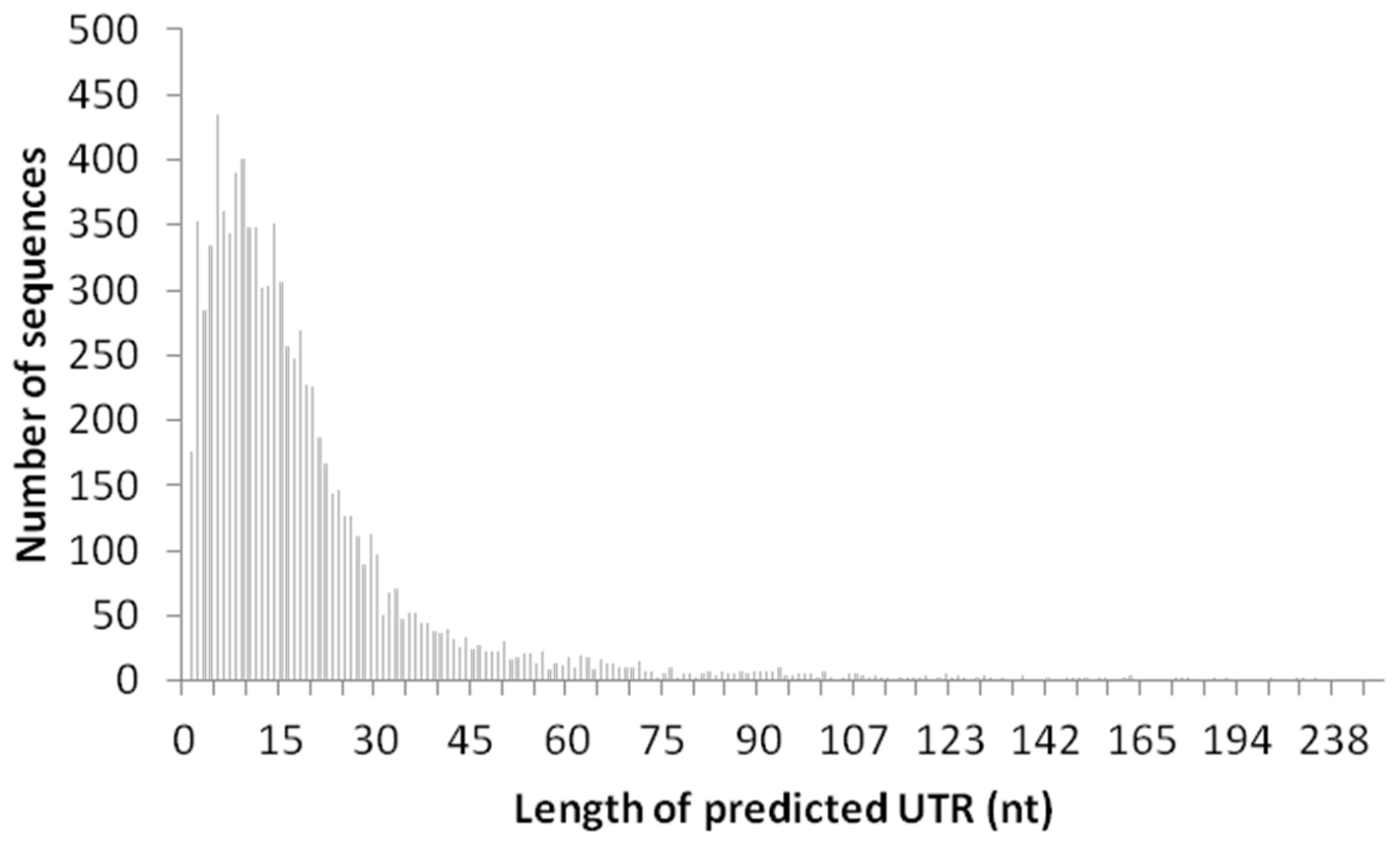
The length distribution of 5′-UTRs of *Eutreptiella* sp. cDNAs. The left skewed distribution pattern indicates high proportion of cDNAs with short 5′-UTRs.

In the 9,004 genes with BLAST hits (E-value ≤10^−3^), the average length of the predicted 5′-UTR was 21±25 nt excluding SL. About 56% of the 5′-UTRs were shorter than 15 nt and 18% <5 nt. The high proportion of short 5′-UTRs is evident in the left skewed histogram (Figure 4). To verify the accuracy of the estimation, we selected 100 genes with significant hit (E-value ≤10^−10^) to the reported genes with known start codon AUG (Figure S3). The estimated 5′-UTR for this subset of genes were 17±16 nt, which was not significantly different (two tailed t-test, p = 0.29) from the bulk estimation. An euglenozoan Kozak-like sequence, AnnAUGnC, where lower case depicts variable sites, n any nucleotide, and upper case conserved nucleotides, was found in some of the genes with significant BLAST hit (Figures S3, S4).

### Diverse Functions Encoded by the *trans*-spliced mRNA Pool

**Figure 5:**
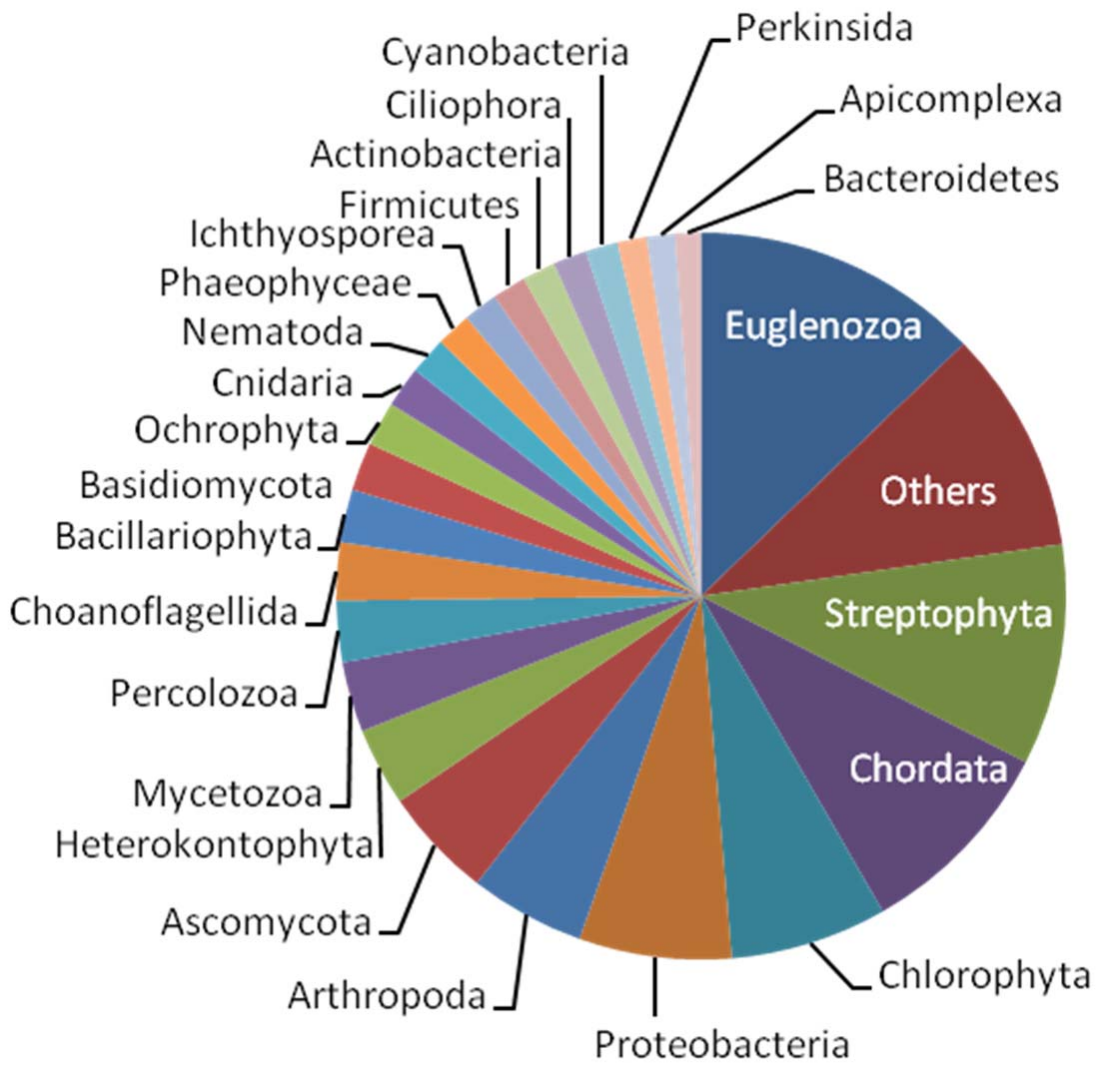
Taxonomic (phylum) distribution of top sequence hits. Phyla that accounted for less than 1% of the unique sequences were pooled as “Others”.

Figure 5 shows the distribution of the top-hit organisms corresponding to the BLAST results. About 13% of the annotated unique sequences showed the top hits to genes of Euglenozoa. The rest of the sequences showed similarity to genes of various other organisms such as streptophytes, chordates, chlorophytes, heterokonts, proteobacteria, arthropods, ascomycetes (5–10% significant matches). Organisms that accounted for less than 1% of the sequences were classified as “other”, which accounted for approximately 10% of the annotated unique sequences.

**Figure 6:**
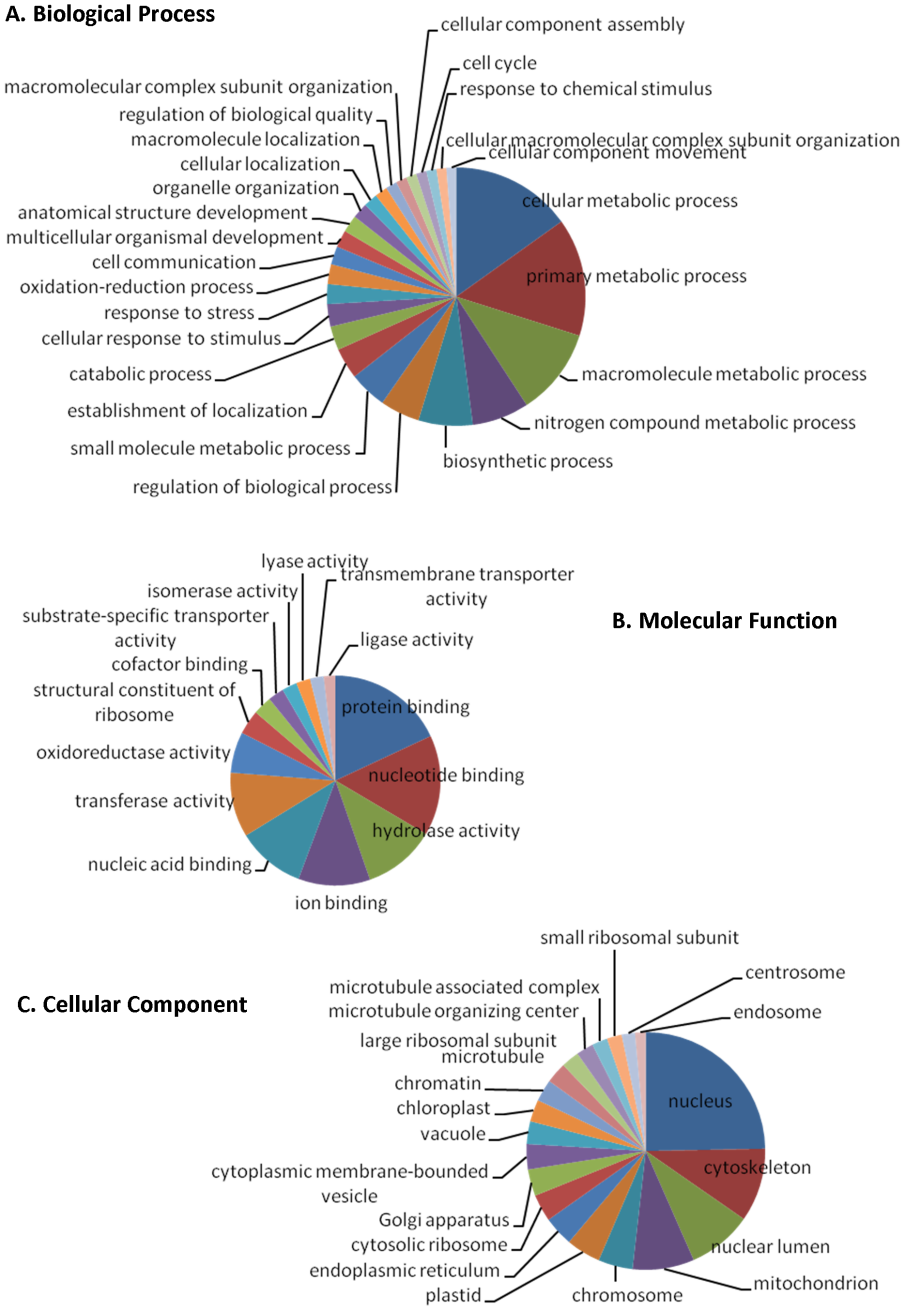
Functional distribution of *Eutreptiella* sp. transcriptomes. The pie charts were generated at level 7 for cellular component and level 3 for biological process and molecular function by using Blast2Go.

Figure 6 shows the distribution of the GO categories related to biological process, molecular function, and cellular component. Protein binding, nucleotide binding, and hydrolase activity are the three major molecular functions that dominated the transcriptomes (Figure 6A). In biological process, approximately half of the transcripts were related to metabolic process (i.e. cellular metabolic, primary metabolic, macromolecule metabolic, and nitrogen compound metabolic processes); the rest were associated with biosynthesis process and variety of other molecular functions (Figure 6B). In cellular component, transcripts potentially related to nucleus, cytoskeleton, nuclear lumen, mitochondrion, chromosome, plastid, and others were identified (Figure 6C).

The *Eutreptiella* sp. transcriptomic dataset represented a wide range of biochemical pathways that have previously been characterized in other organisms. Those pathways included photosynthetic carbon fixation (Table 2), glycolysis, gluconeogenesis, citrate cycle, pentose pathway, pyruvate metabolism, alanine, aspartate and glutamate metabolism, fructose and mannose metabolism, glycine, serine and threonine metabolism, amino sugar and nucleotide sugar metabolism, arginine and proline metabolism, glutathione metabolism, purine metabolism, aminoacyl-tRNA biosynthesis, pantothenate and CoA biosynthesis (Table S2–S14). In the following, we will focus on carbon fixation pathways.

**Table 2:** Candidate genes involved in carbon fixation in *Eutreptiella* sp. compared with other potential C_4_ algae *Thalassiosira pseudonana* (*T.ps*) and *Ostreococcus tauri* (*O.ta*).

Gene	EC number	*Eutreptiella*	*O.ta* c	*T.ps* c
Alanine transaminase	2.6.1.2	1	1	2
Aspartate transaminase^a^	2.6.1.1	3	3	7
Fructose-bisphosphatase	3.1.3.11	1	4	3
Fructose-bisphosphate aldolase	4.1.2.13	4	2	3
Glyceraldehyde-3-phosphate dehydrogenase (NDAP+)	1.2.1.1.13	1	1	0
Malate dehydrogenase (NADP+)^a^	1.1.1.82	1	1	0
Malate dehydrogenase (decarboxylating)^a^	1.1.1.39	–	1	1
Malate dehydrogenase^a^	1.1.1.40	2	2	0
Malate dehydrogenase^a^	1.1.1.37	9	2	2
Phosphoribulokinase	2.7.1.19	1	1	1
Phosphoglycerate kinase	2.7.2.3	8	2	6
Phopsphoenolpyruvate carboxylase^a^	4.1.1.31	1	1	2
Phosphoenolpyruvate carboxykinase^a^	4.1.1.49	4	0	1
Pyruvate kinase^a^	2.7.1.40	1	2	5
Pyruvate, phosphate dikinase^a^	2.7.9.1	1	2	1
Ribulose-biphosphate carboxylase	4.1.2.13	1^b^	6	2
Ribulose-phosphate 3-epimerase	5.1.3.1	1	3	3
Ribose 5-phosphate epimerase	5.3.1.6	–	1	2
Sedoheptulose-bisphosphatase	3.1.3.37	1	1	1
Transketolase	2.2.1.1	4	1	2
Triose-phosphate isomerase	5.3.1.1	6	2	4

The numbers for *Eutreptiella* represents the number of unique sequences found in our dataset. The numbers of genes in other organisms are based on genome annotations. Sequences and annotation results are listed in Table S15.

– Genes were not found in our dataset. This might be due to insufficient sequencing depth.

a Genes potentially involved in C_4_ carbon fixation.

b Obtained from cloning instead of transcriptome.

c Data adapted from Derelle et al.[63].

### Genes Encoding Carbon-fixing Enzymes and CO_2_ Concentrating Mechanism in *Eutreptiella* sp

Enzymes required for C_4_ photosynthesis were identified in *Eutreptiella* sp. (Table 2 and Table S15). The genes in this pathway that we found in *Eutreptiella* sp. cDNA libraries included: aspartate transaminase (EC 2.6.1.1), NADP dependent malate dehydrogenase (EC 1.1.1.82), malate dehydrogenase (EC 1.1.1.40, EC 1.1.1.37), phosphoenolpyruvate carboxylase (PEPCase, EC 4.1.1.31), phosphoenolpyruvate carboxykinase (EC 4.1.1.49), pyruvate kinase (EC 2.7.1.40), and pyruvate phosphate dikinase (EC 2.7.9.1). Among these genes, PEPCase is also known to function in non-photosynthetic CO_2_ fixation in euglenoids ([43] and refs therein). We also identified most of the genes involved in Calvin-Benson cycle: fructose bisphosphatase (EC 3.1.3.11), fructose-bisphosphate aldolase (EC 4.1.2.13), glyceraldehyde-3-phosphate dehydrogenase (EC 1.2.1.1.13), phosphoribulokinase (EC 2.7.1.19), phosphoglycerate kinase (EC 2.7.2.3), ribulose-phosphate 3-epimerase (EC 5.1.3.1), sedoheptulose-bisphosphatase (EC 3.1.3.37), transketolase (EC 2.2.1.1), triose-phosphate isomerase (EC 5.3.1.1). However, ribulose-3, 5 bisphosphate carboxylase/oxygenase (RuBisCo, EC 4.1.2.13) and ribose 5-phosphate epimerase (EC 5.1.3.6) were missing in the 454 dataset (Table 2). In addition, eight unique sequences were identified as carbonic anhydrase (Table S15), which may function as the carbon concentrating mechanism in algae.

## Discussion

### Widespread *Trans*-splicing in *Eutreptiella* sp

If SL *trans*-splicing is limited to some genes, the SL-based cDNA libraries would only cover a fraction of the complete transcriptome of this species and a limited functional (proteomic) diversity would be expected. However, several lines of evidence emerging from our dataset indicate that it is not the case. Clustering of our SL-based 454 transcriptome reads yielded 36,643 unique transcripts (unique genes). In addition, the annotated genes from our SL-based transcriptomes specify very diverse functions (Figure 6, Tables S2–S14).

Among the genes in the typical Calvin-Benson cycle, the gene encoding RuBisCo large subunit (*rbcL*) and ribose 5-phosphate epimerase were missing in the SL-based 454 libraries (Table 2). However, using PCR with a *rbcL* universal primer set [44], we were able to isolate *rbcL* cDNA from *Eutreptiella* sp. (GenBank Accession number JX478224). *RbcL* is typically encoded in the chloroplast genome of eukaryotic algae except dinoflagellates, which harbor the gene in the nuclear genome [23]. Further, a BLAST search with the plastid-encoded genes reported in *Eutreptiella gymnastica* [45] as the query against our *Eutreptiella* sp. 454 transcriptome dataset only yield ferredoxin NADP+ reductase. Failure to find this and the transcripts of most of the other plastid protein-coding genes in our SL-based *Eutreptiella* sp. 454 transcriptome dataset could be due to these plastid genes are not SL *trans*-spliced and thus absent in the cDNA libraries prepared using Eut-SL as a selective primer, or due to plastid-targeted proteins in this species have long leader sequences and the sequences we got for these genes could not have significant hits to homologs in GenBank. However, in dinoflagellates, SL *trans*-splicing is also widespread among the nucleus-encoded mRNAs, but not plastid-encoded mRNAs [23]. The current finding of SL *trans*-splicing pattern possibly adds one more element in support of the notion that Euglenozoa and Dinoflagellata share genetic features likely as a result of converging evolution [46].

From the *Eutreptiella* sp. 454 dataset, 36,643 unique genes were identified. The rarefaction curve built on the 454 dataset indicated that our sequencing depth was not exhaustive relative to the number of genes in the cDNA libraries and more genes are expected (Figure S2). There is no evidence that *Eutreptiella* sp. or related species have polyploidy a genome. Even if the cultures are diploid, it seems that gene number in *Eutreptiella* sp. is still higher than that in other documented Euglenozoa species, which are all kinetoplastid parasites. In GenBank, 14 Euglenozoa species have complete genome sequences and only 5 out of the 14 species have the data of the total gene numbers. The 5 kinetoplastid parasites are: *Trypanosoma cruzi*, *Trypanosoma brucei, Leishmania major, Leishmania braziliensis,* and *Leishmania infantum* [47], [48], [49]. Among these species, *T. cruzi* contains the highest number of protein coding genes, ∼12,000 genes/haploid cell [47]. As parasites, their genome sizes could have been reduced remarkably compared to free living Euglenozoa lineages and relatively smaller than the free-living *Eutreptiella*. Although currently there is no complete genome data of free-living Euglenozoa for a comparison, studies have shown gene deletion occurs in parasitic Euglenozoa [47]. In our study, 38 gene families had more than 10 copies of genes (Table S1). The alignment of histone H2A illustrates multiple copies (Figure S5). The high gene number of *Eutreptiella* sp. is likely to represent a functionally redundant proteome (i.e. widespread gene duplication) as is presumably the case for dinoflagellates [50].

### Unique Transcriptome and Great Number of Novel Genes

In our dataset, the sequences with top BLAST hit matched very diverse groups of organisms reported in the public databases. To avoid any source of contamination into our dataset, we had carefully designed and carried out the entire study: 1) the cultures were raised in a place separated from other algal cultures; 2) 18S rRNA gene was PCR amplified from the DNA of the cultures and directly sequenced to confirm that there was only one clean sequence identical to our previous report of 18S rRNA of this species; 3) mRNA was extracted from total RNA using oligo-dT based method to eliminated bacteria DNA contamination; 4) *Eutreptiella*-specific Eut-SL primer was used to construct all the libraries for 454 sequencing to avoid any possible contaminants during library construction; 5) DeconSeq program was used to eliminate any possible sequences from bacteria or human contamination. Although we cannot entirely exclude the possibility, it is very unlikely that our *Eutreptiella* sp. 454 dataset contains contaminant genes from other organisms.

Although the highest percentage (13%) of the sequences had Euglenozoa as the top-hit source organisms, this ratio is much lower than that of the transcriptomic data of a green alga *Dunaliella tertiolecta* to its related linage (59% of sequences has Chlorophyceae to be the top-hit source organisms) [51]. Among euglenoid algae, many studies have focused on the genus *Euglena*, but none of them involved large-scale transcriptomic sequencing. The limited transcriptomic data from the euglenoids may explain why as high as 72% of the unique sequences had no matches to documented genes. In other studies, more than 60% of unknown genes is not unusual in other transcriptomic research on marine organisms (e.g. 65% in cnidarians [52]). To evaluate if the high number of unknown genes was due to annotation methodology, we annotated the data again by different methods. FragGeneScan, which was designed for finding genes in short reads [53], was used to predict open reading frames in our 454 clusters; then the candidate sequences were searched against hidden Markov models (HMMs) [54] by using Pfam HMMS [55] and TIGRFAMS [56]. Still 74% of the genes remained unknown, which is similar to the results of Blast2Go. It is possible that the functional domains in *Eutreptiella* are unique and not identifiable using the HMMS in Pfam or that our 454 reads are not long enough to reach the domains of the genes in the alga. While only 28% of the unique cDNA sequences we retrieved showed significant similarity to documented genes, this small portion encodes a functionally diverse set of genes, as indicated in the previous section.

Furthermore, when we examined unknown genes based on different expression levels, we found that a large portion (80%) of the singletons were unknown genes (Figure 3). These unknown low-abundance transcripts may represent rare genes that usually express at low levels or transiently, so they were never identified or studied previously. In contrast, highly expressed genes (represented by >3000 reads in our libraries) were more annotatable with only 37% without significant hits. As highly expressed genes may have important biological functions, the functions of these unknown genes should be characterized in the future. Our first *Eutreptiella* sp. large dataset, with a great number of potentially novel genes, will constitute a valuable genetic resource for future genomic studies on euglenoids.

Potentially, the unknown sequences could be gene fragments in the middle of larger transcripts; however, the likelihood is very low because 1) all our cDNA sequences were obtained by use of the Eut-SL sequence, which is specifically *trans-*spliced to the 5′-end of the mRNAs, as demonstrated previously in other Euglenoids (e.g. [17]); 2) when Eut-SL was used in the single primer negative control, no visible cDNAs were detected by agarose gel electrophoresis (Figure 2), excluding the possibility of nonspecific amplification of cDNA fragments with this sequence as the sole primer; 3) for the sequences with significant hit to the reported genes (e.g. Figure S3), the Eut-SL is clearly located at the very 5′-end of the cDNA.

### Short 5′-UTRs

Most of the mRNAs in *Eutreptiella* sp. seem to have short 5′-UTRs, as demonstrated by transcriptome-wide and selected-gene analyses (Figures 4 and S3). The short 5′-UTRs may be related to the translation regulation in *Eutreptiella* sp. At the initiation step of translation, two subunits of the ribosome bind to the 5′-cap of the mRNA at and walk through the 5′-UTR to AUG start codon. Elements in the 5′-UTR can control mRNA translation via various mechanisms, such as secondary structures, binding sites for regulatory proteins or RNA, and internal ribosome entry sites [57], [58]. Some other eukaryotes also have short 5′-UTRs in their mRNAs. For instance, it has been reported that in the mRNAs of chordate *Okopleura dioca* the average distance from the spliced leader to the predicted start codon for 90 transcripts was 22 nt [59]. The lengths of 5′-UTRs are related to translation efficiency [60]. In general, 5′-UTRs that are short, relatively unstructured, and have lower GC content enable efficient translation [57], [60]. In some cases, the 5′-UTRs of mRNAs in *Eutreptiella* sp. are only a few nucleotides long. About 18% of the mRNAs in *Eutreptiella* sp. were predicted to have 5′-UTRs shorter than 5 nt. For instance, the 5′-UTR of ubiquitin-activating enzyme transcript has 5 nt (UCACC). The short and unstructured 5′-UTRs may be a result of SL *trans*-splicing and may facilitate efficient translation of these mRNAs. Interesting, Kozak-like sequences conserved in the mRNAs of Euglenozoa, land plants, and other eukaryotes also occur in some of the transcripts of *Eutreptiella* sp., suggesting similar translation regulation between *Eutreptiella* sp. and other organisms. However, a substantial number of sequences in *Eutreptiella* sp. do not seem to contain these sequences, suggesting possible presence of unique Kozak-like sequences in this alga, which should be studied further in the future.

Practically, the short 5′-UTR in *Eutreptiella* sp. could be beneficial to transcriptome research. As demonstrated in this study, instead of using a traditional 454 sequencing method, the spliced leader can be used as a primer in cDNA preparation to obtain 5′ transcriptome information. The short 5′-UTR allows sequencing reads to cover longer coding regions, thus enhancing the success of gene annotation.

Although our data indicate that the *Eutreptiella* sp. transcripts with GenBank hits have short 5′-UTR, there is a possibility that some transcripts have so long 5′-UTRs that they cause no significant hit to the sequences in the public databases. This remains to be addressed in the future by sequencing the complete transcripts of *Eutreptiella* sp.

### Potential Complex Carbon Fixation Mechanisms in *Eutreptiella* sp

**Figure 7:**
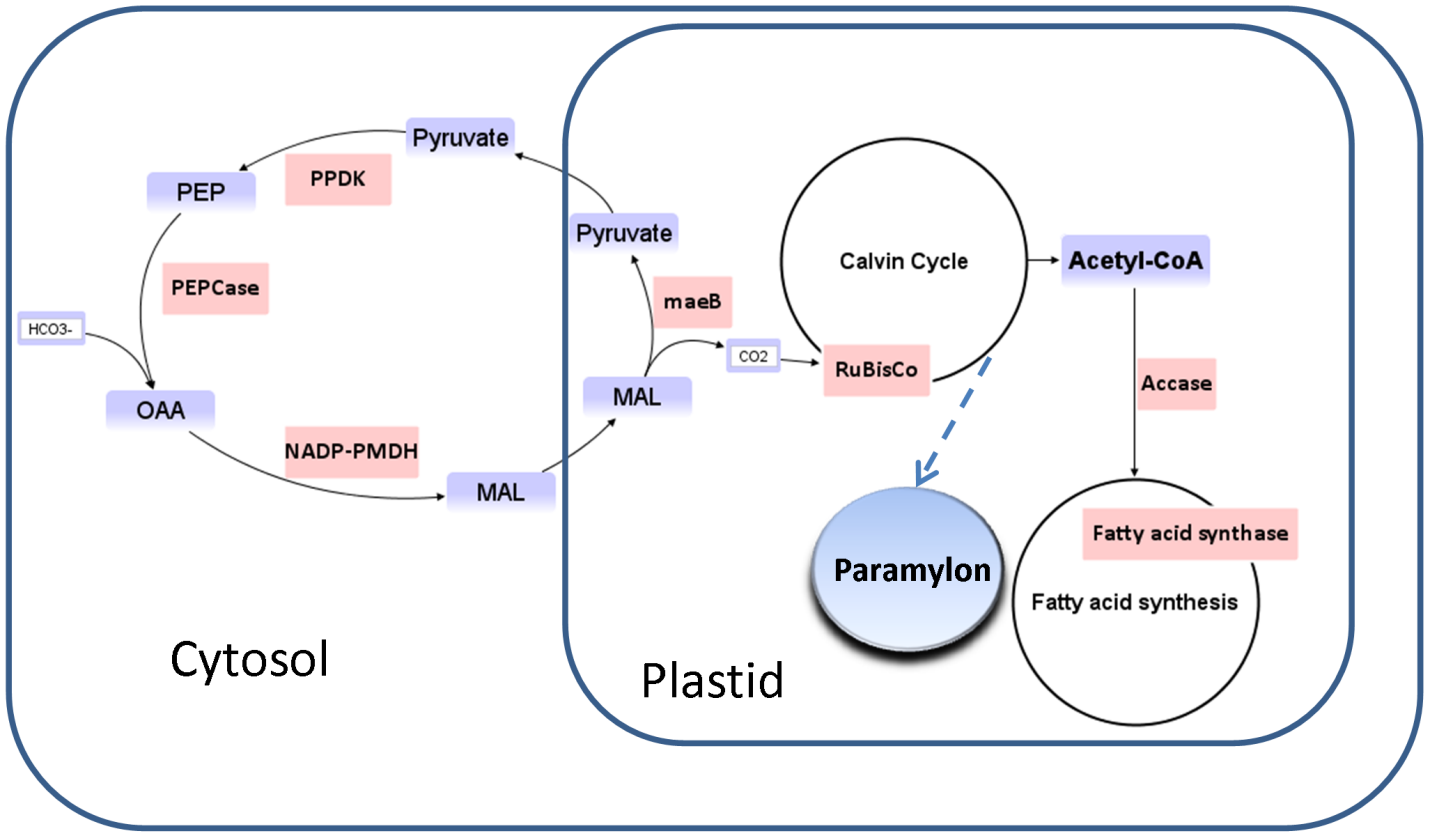
Hypothetical C_4_ pathway for CO_2_ fixation in *Eutreptiella* sp. PEP: phosphoenolpyruvate; OAA: oxaloacetate; MAL: malate; PEPCase: phosphoenolpyruvate carboxylase; PPDK: pyruvate-phosphate dikinase; NADP-PMDH: malate dehydrogenase (NADP+); maeB: malate dehydrogenase (oxaloacetate-decarboxylating)(NADP+). Enzymes are highlighted in pink. Modified from Riebesell [74].

Our transcriptomic data and literature information from the related organisms suggest that *Eutreptiella* sp. may possess a complex carbon acquisition and fixation system. First, our *Eutreptiella* sp. transcriptomic data reveal genes involved in C_4_ carbon fixation documented in other organisms. All but one (ribose 5-phosphate epimerase) of the genes essential for both C_4_ and C_3_ carbon fixation pathways were found in our *Eutreptiella* transcriptomic dataset (Table 2). The unicellular 4-carbon cycle has been considered as a CO_2_-concentrating mechanism in some microalgae, such as the diatom *Thalassiosira weissflogii* [61], [62] and the green alga *Ostreococcus tauri* [63]. In higher plants, C_4_ carbon fixation and RuBisCo activity are spatially separated by different types of cells (i.e. mesophyll cells and bundle-sheath cells [64]). Unicellular organisms have been hypothesized to utilize different intracellular compartments for C_4_ photosynthesis [65]. In the diatom *T. weissflogii*, PEPCase is postulated to catalyze bicarbonate to phosphoenolpyruvate (PEP) to form 4-carbon oxaloacetate in the cytoplasm. Presumably, the oxaloacetate is then transported into the chloroplast and reduced to malate by NADP^+^ malate dehydrogenase. The malate is oxidized and decarboxylated to pyruvate and CO_2_ by pyruvic-malic carboxylase. The CO_2_ is then fixed by RuBisCo. The pyruvate is transferred back to the cytoplasm and is converted to PEP by pyruvate phosphate dikinase. Based on the information available and the genes found in this study, a potential C_4_ fixation pathway and downstream metabolism scheme can be deduced for *Eutreptiella* sp. (Figure 7). Second, euglenoids have been shown to be able to take up organic compounds and perform heterotrophic carbon fixation under light [66], [67] and dark [43], [67], [68]. The heterotrophic carbon fixation involves PEPCase and phosphoenolpyruvate carboxykinase (PEPCK), which have been demonstrated in *Euglena*, and cooperation between PEPCase and RuBisCo in photosynthetic CO_2_ fixation may actually occur [66]. When carbon substrates (e.g. lactate [43], [67]) are provided, *Euglena gracilis* can fix carbon in the presence of gluconeogenesis, mechanisms similar to those described in animal cells [43]. Earlier studies using ^14^C isotopes indicated that PEPCase and PEPCK were associated with *Euglena* heterotrophic carbon fixation [43], [67] to synthesize a linear (1–3)-β-d-glucan, paramylon, which is an unique intracellular storage carbohydrate in euglenoids [69], [70]. Briand et al. [43] reported that ^14^C incorporation was found in malate, aspartate, citrate, PEP, 3-phosphoglyceric acid (PGA), fructose 1,6 biphosphate, and sugar monophosphates. In a previous study, we found that *Eutreptiella* sp. can grow in f/2 medium under continuous dark for 7 days [18]. The culture grew 16-fold from the initial concentration (from about 2,800 cells/ml to 46,000 cells/ml), with no evidence of ingesting bacteria to support their growth [18]. Hence, this species may be able to perform heterotrophic carbon fixation under dark conditions as in *Euglena gracilis* [43].

Third, carbonic anhydrase (CA) was found in our *Eutreptiella* transcriptomic dataset (Table S15), which may function as the carbon concentrating mechanism (CCM). Carbonic anhydrase exists in many photosynthetic organisms in which it catalyzes the conversion between bicarbonate and CO_2_ [71]. However, several types of CAs are localized in various cellular locations and play different functional roles [72]. One of the eight unique sequences found in *Eutreptiella* sp. was similar to delta-CA in *Lingulodinium polyedrum* (58% of nucleotide similarity), which helps to increase CO_2_ availability [73], suggesting that *Eutreptiella* sp. may use CA as their CCM.

With all of these mechanisms, *Eutreptiella* sp. has the potential to switch strategies for carbon acquisition and fixation under different environmental conditions. Hence, the source of PGA, which is the material for synthesizing paramylon in *Eutreptiella* can potentially be produced via three ways: (1) when there is sufficient light and CO_2_, PGA is synthesized in the Calvin-Benson Cycle in chloroplast via C_3_ photosynthesis; (2) under dark conditions, heterotrophic carbon fixation can occur and PGA is synthesized via gluconeogenesis in mitochondria; (3) when CO_2_ is not sufficient, C_4_ pathway or CAs are involved to concentrate CO_2_ for RuBisCo to fix. A key to verifying this hypothesis is to determine the role of the enzymes, particularly PEPCase. Whether it is only responsible for heterotrophic carbon fixation or is involved in the C_4_ pathway or both in *Eutreptiella* sp. deserves some focused research effort.

## Supporting Information

Figure S1 **The coverage-identity plot of the detection of contaminations by DeconSeq** [**26**] **.**
(TIF)

Figure S2 **Rarefaction curves of the 454 sequencing reads.** The end point of the assimilation is 1,648,000 genes. Additional 3.6 genes were found when the sequence reads increased from 1,647,500 to 1,648,000, which was the stop point of the assimilation.
(TIF)

Figure S3 **Examples of the start codon ATG and the flanking nucleotides of** ***Eutreptiella*** **sp. transcripts having significant BLASTx hit to genes with conserved ATG.**
(TIF)

Figure S4 **The flanking nucleotides of the prediction start codon AUG for the 100 genes with significant BLASTx hit to the genes with conserved start codon.** The A of AUG is numbered as +1; X at positions −4 to −2 indicates the missing nucleotide in some transcripts at that position. Logos were created using online program WebLogo (http://weblogo.berkeley.edu/logo.cgi) under Frequency Plot mode.
(TIF)

Figure S5 **The alignment of paralogs of histone h2a.**
(TIF)

Table S1 **Genes or gene families that contained more than 10 paralogs.**
(DOCX)

Table S2 **Candidate genes involved in citrate cycle.**
(DOCX)

Table S3 **Candidate genes involved in glycolysis/gluconeogenesis.**
(DOCX)

Table S4 **Candidate genes involved in pentose phosphate pathway.**
(DOCX)

Table S5 **Candidate genes involved in alanine, aspartate and glutamate metabolism.**
(DOCX)

Table S6 **Candidate genes involved in fructose and mannose metabolism.**
(DOCX)

Table S7 **Candidate genes involved in glycine, serine and threonine metabolism.**
(DOCX)

Table S8 **Candidate genes involved in amino sugar and nucleotide sugar metabolism.**
(DOCX)

Table S9 **Candidate genes involved in arginine and proline metabolism.**
(DOCX)

Table S10 **Candidate genes involved in glutathione metabolism.**
(DOCX)

Table S11 **Candidate genes involved in pyruvate metabolism.**
(DOCX)

Table S12 **Candidate genes involved in purine metabolism.**
(DOCX)

Table S13 **Candidate genes involved in aminoacyl-tRNA biosynthesis.**
(DOCX)

Table S14 **Candidate genes involved in pantothenate and CoA biosynthesis.**
(DOCX)

Table S15 **Sequences and annotation results of the candidate genes involved in carbon fixation and the carbon concentrating mechanism (CCM) in** ***Eutreptiella*** **sp.**
(DOCX)
