# Supplementary figures and images for: Transcriptomic Study Reveals Widespread Spliced Leader Trans-Splicing, Short 5′-UTRs and Potential Complex Carbon Fixation Mechanisms in the Euglenoid Alga Eutreptiella sp
Source: PLoS One. 2013 Apr 9;8(4):e60826. doi: 10.1371/journal.pone.0060826 (PMC3621762; doi:10.1371/journal.pone.0060826)

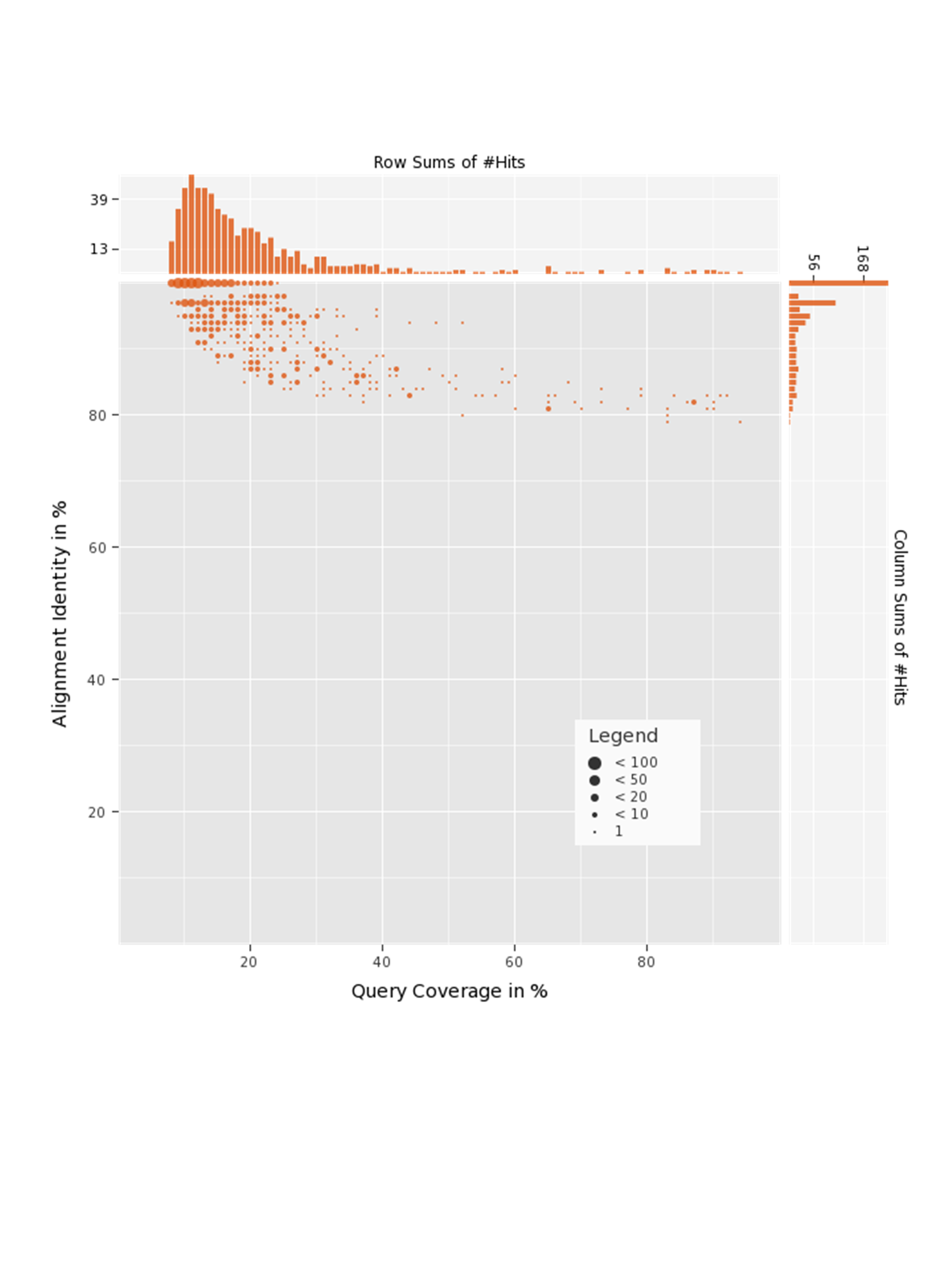

Supplement: Figure S1 — The coverage-identity plot of the detection of contaminations by DeconSeq [26] . (TIF) [file pone.0060826.s001.tif]

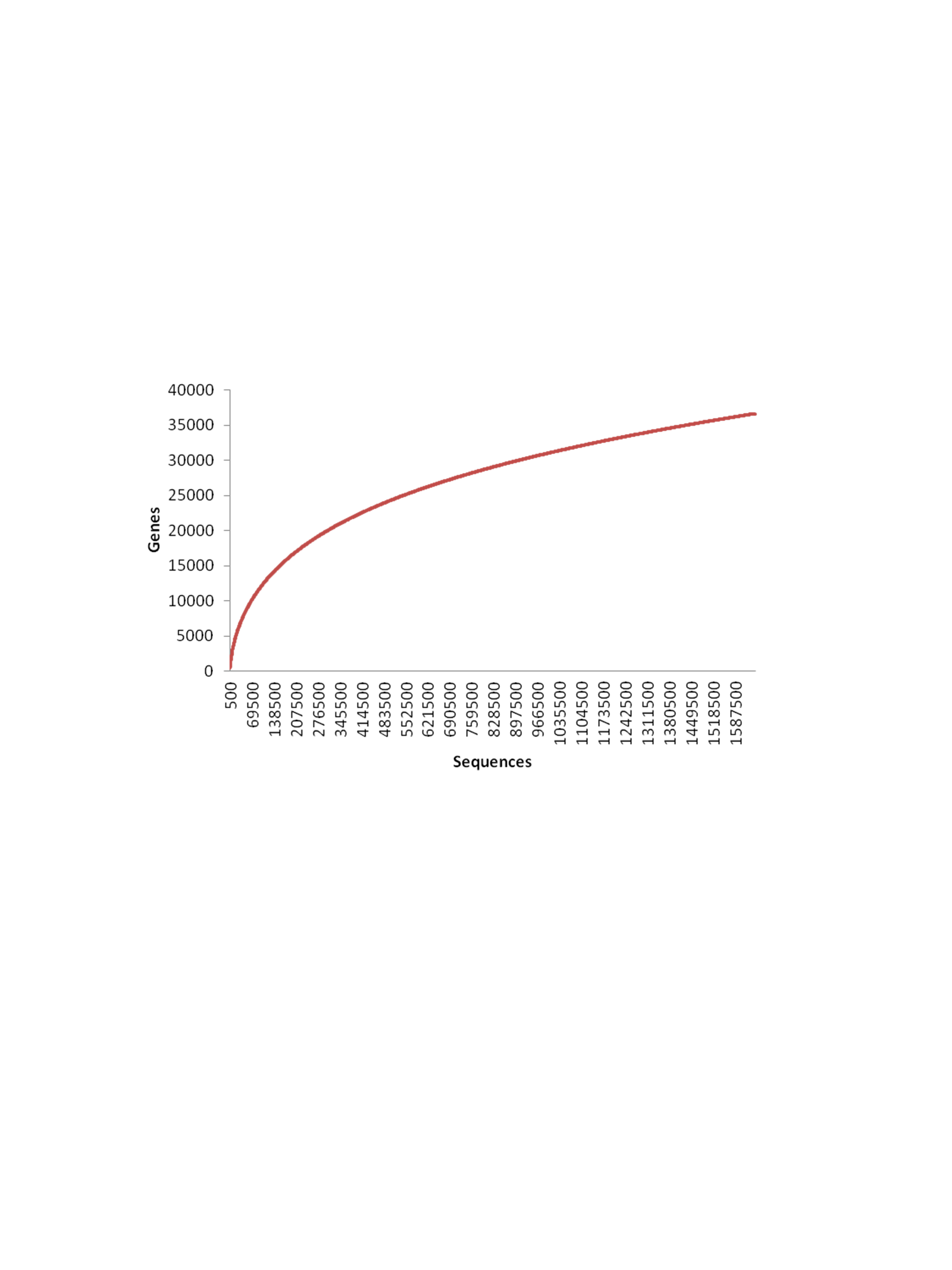

Supplement: Figure S2 — Rarefaction curves of the 454 sequencing reads. The end point of the assimilation is 1,648,000 genes. Additional 3.6 genes were found when the sequence reads increased from 1,647,500 to 1,648,000, which was the stop point of the assimilation. (TIF) [file pone.0060826.s002.tif]

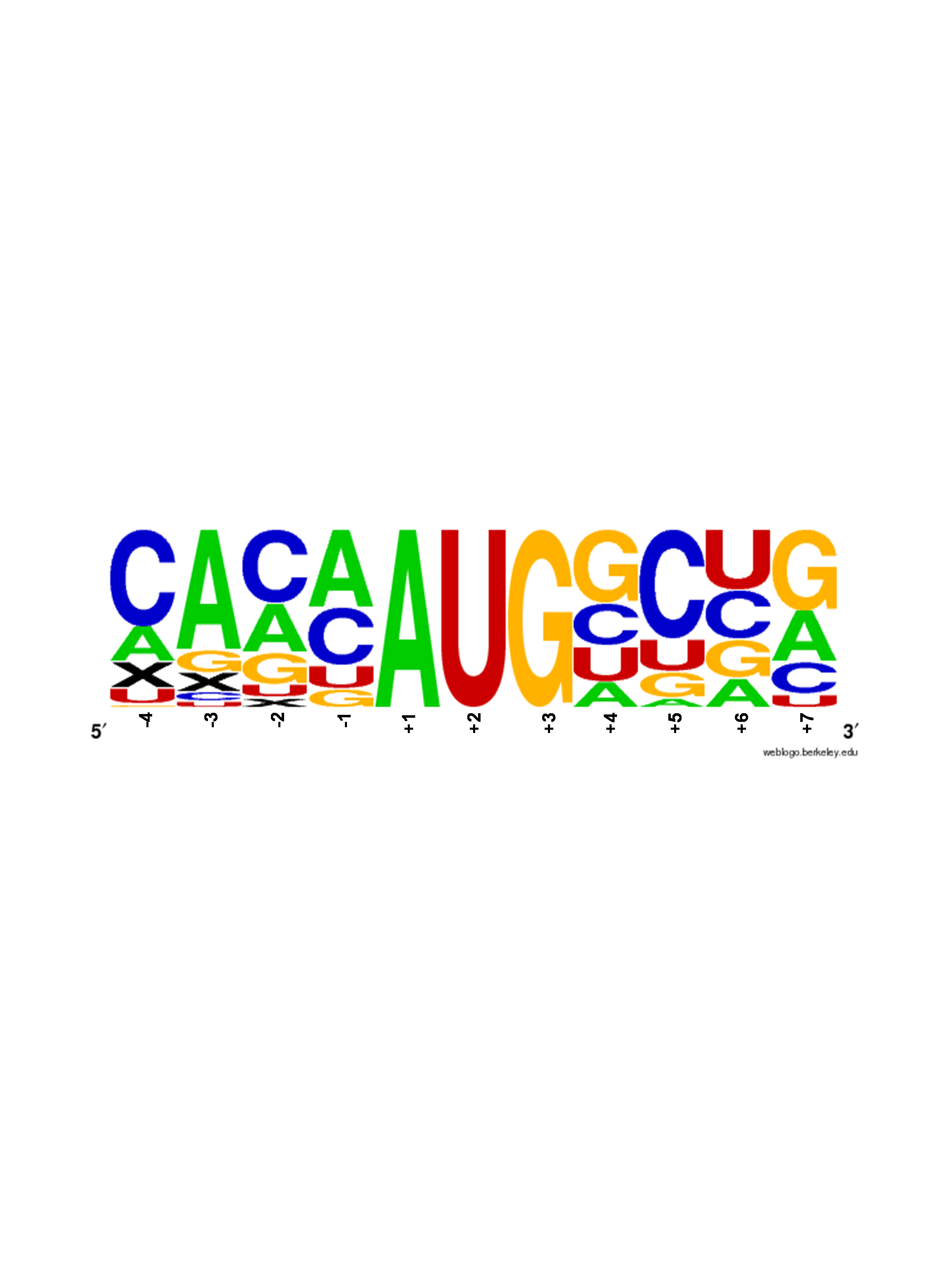

Supplement: Figure S4 — The flanking nucleotides of the prediction start codon AUG for the 100 genes with significant BLASTx hit to the genes with conserved start codon. The A of AUG is numbered as +1; X at positions −4 to −2 indicates the missing nucleotide in some transcripts at that position. Logos were created using online program WebLogo (http://weblogo.berkeley.edu/logo.cgi) under Frequency Plot mode. (TIF) [file pone.0060826.s004.tif]

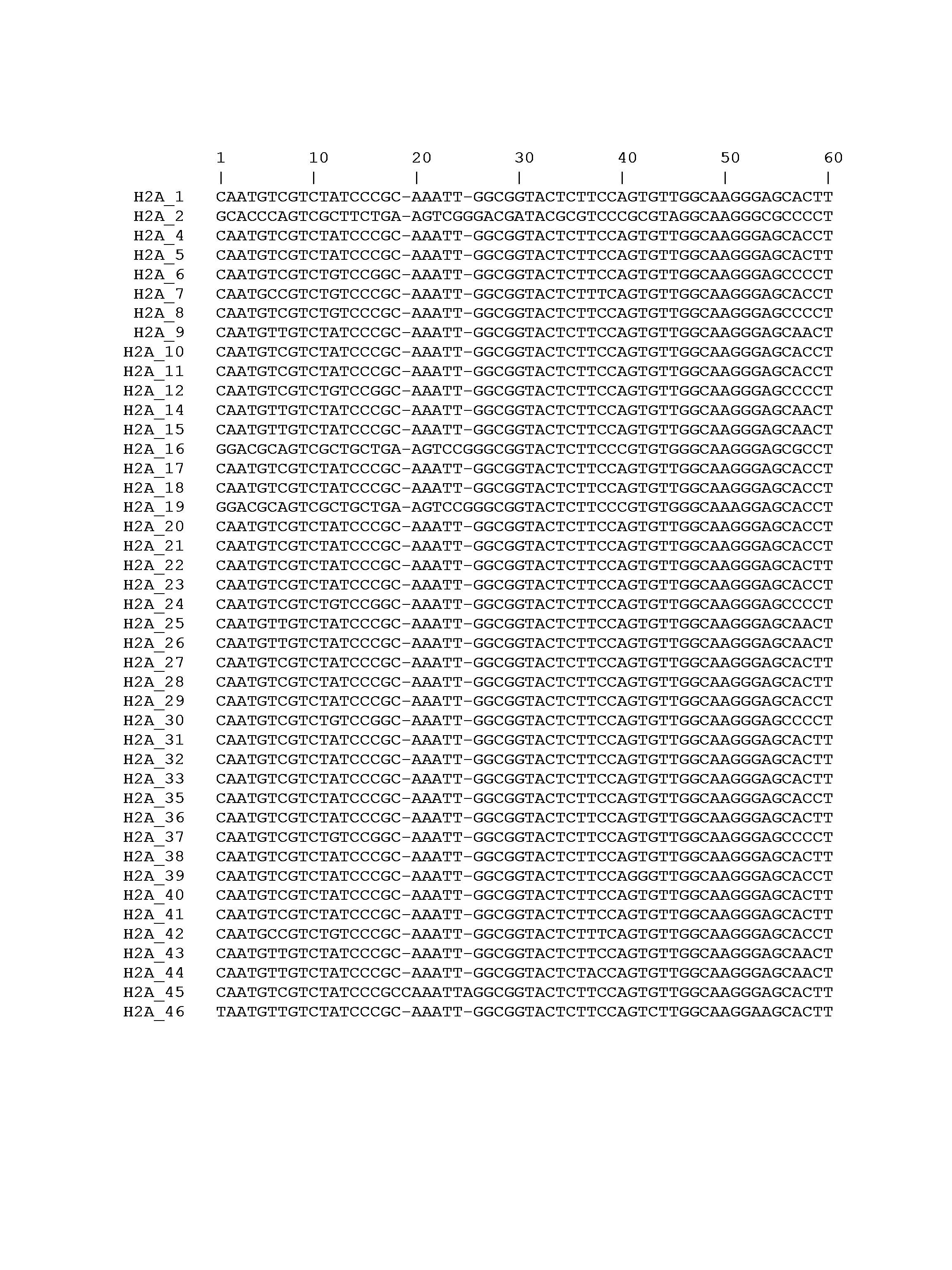

Supplement: Figure S5 — The alignment of paralogs of histone h2a. (TIF) [file pone.0060826.s005.tif]
