# Supplementary material for: Transcriptomic Study Reveals Widespread Spliced Leader Trans-Splicing, Short 5′-UTRs and Potential Complex Carbon Fixation Mechanisms in the Euglenoid Alga Eutreptiella sp
Source: PLoS One. 2013 Apr 9;8(4):e60826. doi: 10.1371/journal.pone.0060826 (PMC3621762; doi:10.1371/journal.pone.0060826)
Supplement: Table S1 — Genes or gene families that contained more than 10 paralogs. (DOCX) [file pone.0060826.s006.docx]

Table S1. Genes or gene families that contained more than 10 paralogs.

| Gene/gene family | Number of transcripts |
| --- | --- |
| histone h2a | 44 |
| 40s ribosomal protein s26 | 18 |
| nad-dependent epimerase dehydratase | 22 |
| peptidyl-prolyl cis-trans isomerase | 22 |
| 60s ribosomal protein l11 | 19 |
| 60s ribosomal protein l26 | 19 |
| 69 kda paraflagellar rod protein | 19 |
| 60s ribosomal protein l37 | 17 |
| kinetoplastid membrane protein kmp-11 | 17 |
| ankyrin unc44 | 16 |
| serine threonine protein kinase | 16 |
| cytochrome c | 15 |
| elongation factor 1- | 15 |
| alpha tubulin | 14 |
| calmodulin | 14 |
| protein disulfide isomerase | 14 |
| 14-3-3 protein | 13 |
| acyl- dehydrogenase | 13 |
| glutathione s-transferase | 13 |
| methyltransferase type 11 | 13 |
| ubiquitin | 13 |
| acetyl- acetyltransferase | 12 |
| adenylate kinase | 12 |
| c2 domain containing protein | 12 |
| cold shock protein | 12 |
| 40s ribosomal protein s8 | 11 |
| 40s ribosomal protein s9 | 11 |
| asparagine synthetase | 11 |
| chain nmr-structure of tryparedoxin 1 | 11 |
| fructose-bisphosphate aldolase | 11 |
| adp-ribosylation factor | 10 |
| diguanylate cyclase with gaf sensor | 10 |
| dihydroxy-acid dehydratase | 10 |
| duf614 family protein | 10 |
| dynein light chain cytoplasmic | 10 |
| eukaryotic translation initiation factor 4e | 10 |
| thioredoxin | 10 |
| zinc finger protein | 10 |
