# Supplementary material for: Transcriptomic Study Reveals Widespread Spliced Leader Trans-Splicing, Short 5′-UTRs and Potential Complex Carbon Fixation Mechanisms in the Euglenoid Alga Eutreptiella sp
Source: PLoS One. 2013 Apr 9;8(4):e60826. doi: 10.1371/journal.pone.0060826 (PMC3621762; doi:10.1371/journal.pone.0060826)
Supplement: Table S2 — Candidate genes involved in citrate cycle. (DOCX) [file pone.0060826.s007.docx]

Table S2. Candidate genes involved in citrate cycle.

| **Gene** | **EC number** | **Number of unique transcripts** |
| --- | --- | --- |
| Aconitate hydratase | 4.2.1.3 | 6 |
| Fumarate hydratase | 4.2.1.2 | 2 |
| Pyruvate dehydrogenase (acetyl-transferring) | 1.2.4.1 | 3 |
| Succinate dehydrogenase | 1.3.99.1 | 3 |
| Succinate-CoA ligase (ADP-forming) | 6.2.1.5 | 3 |
| Succinate-CoA ligase (GDP-forming) | 6.2.1.4 | 3 |
| Dihydrolipoyl dehydrogenase | 1.8.1.4 | 1 |
| Citrate (pro-3S)-lyase | 4.1.3.6 | 1 |
| Dihydrolipoyllysine-residue acetyltransferase | 2.3.1.12 | 1 |
| Isocitrate dehydrogenase (NADP+) | 1.1.1.42 | 3 |
| Malate dehydrogenase | 1.1.1.37 | 6 |
| Succinate dehydrogenase (ubiquinone) | 1.3.5.1 | 3 |
| ATP citrate synthase | 2.3.3.8 | 3 |
| Citrate (Si)-synthase | 2.3.3.1 | 5 |
