# Supplementary material for: Transcriptomic Study Reveals Widespread Spliced Leader Trans-Splicing, Short 5′-UTRs and Potential Complex Carbon Fixation Mechanisms in the Euglenoid Alga Eutreptiella sp
Source: PLoS One. 2013 Apr 9;8(4):e60826. doi: 10.1371/journal.pone.0060826 (PMC3621762; doi:10.1371/journal.pone.0060826)
Supplement: Table S3 — Candidate genes involved in glycolysis/gluconeogenesis. (DOCX) [file pone.0060826.s008.docx]

Table S3. Candidate genes involved in glycolysis/gluconeogenesis.

| **Gene** | **EC number** | **Number of unique transcripts** |
| --- | --- | --- |
| 6-phosphofructokinase | 2.7.1.11 | 1 |
| Pyruvate dehydrogenase (acetyl-transferring) | 1.2.4.1 | 3 |
| Glucokinase | 2.7.1.2 | 1 |
| Phosphopyruvate hydratase | 4.2.1.11 | 17 |
| Acetate-CoA ligase | 6.2.1.1 | 3 |
| Dihydrolipoyl dehydrogenase | 1.8.1.4 | 1 |
| Bisphosphoglycerate mutase | 5.4.2.4 | 1 |
| Phosphoglucomutase | 5.4.2.2 | 2 |
| Phosphoglycerate mutase | 5.4.2.1 | 1 |
| Fructose-bisphosphatase | 3.1.3.11 | 2 |
| Dihydrolipoyllysine-residue acetyltransferase | 2.3.1.12 | 1 |
| Glyceraldehyde-3-phosphate dehydrogenase (phosphorylating) | 1.2.1.12 | 8 |
| Fructose-bisphosphate aldolase | 4.1.2.13 | 5 |
| Glucose-6-phosphate isomerase | 5.3.1.9 | 1 |
| Triose-phosphate isomerase | 5.3.1.1 | 7 |
| Polyphosphate-glucose phosphotransferase | 2.7.1.63 | 1 |
| Phosphoglycerate kinase | 2.7.2.3 | 8 |
| Pyruvate kinase | 2.7.1.40 | 1 |
