# Supplementary material for: Transcriptomic Study Reveals Widespread Spliced Leader Trans-Splicing, Short 5′-UTRs and Potential Complex Carbon Fixation Mechanisms in the Euglenoid Alga Eutreptiella sp
Source: PLoS One. 2013 Apr 9;8(4):e60826. doi: 10.1371/journal.pone.0060826 (PMC3621762; doi:10.1371/journal.pone.0060826)
Supplement: Table S4 — Candidate genes involved in pentose phosphate pathway. (DOCX) [file pone.0060826.s009.docx]

Table S4. Candidate genes involved in pentose phosphate pathway.

| **Gene** | **EC number** | **Number of unique transcripts** |
| --- | --- | --- |
| Ribokinase | 2.7.1.15 | 1 |
| 6-phosphofructokinase | 2.7.1.11 | 1 |
| 6-phosphogluconolactonase | 3.1.1.31 | 1 |
| Ribulose-phosphate 3-epimerase | 5.1.3.1 | 1 |
| Glucose oxidase | 1.1.3.4 | 1 |
| Phosphoglucomutase | 5.4.2.2 | 2 |
| Transaldolase | 2.2.1.2 | 1 |
| Transketolase | 2.2.1.1 | 4 |
| Fructose-bisphosphatase | 3.1.3.11 | 2 |
| Glucose-6-phosphate dehydrogenase | 1.1.1.49 | 1 |
| Phosphogluconate dehydrogenase (decarboxylating) | 1.1.1.44 | 3 |
| Fructose-bisphosphate aldolase | 4.1.2.13 | 5 |
| Glucose-6-phosphate isomerase | 5.3.1.9 | 1 |
| Ribose-phosphate diphosphokinase | 2.7.6.1 | 1 |
