# Supplementary material for: Transcriptomic Study Reveals Widespread Spliced Leader Trans-Splicing, Short 5′-UTRs and Potential Complex Carbon Fixation Mechanisms in the Euglenoid Alga Eutreptiella sp
Source: PLoS One. 2013 Apr 9;8(4):e60826. doi: 10.1371/journal.pone.0060826 (PMC3621762; doi:10.1371/journal.pone.0060826)
Supplement: Table S5 — Candidate genes involved in alanine, aspartate and glutamate metabolism. (DOCX) [file pone.0060826.s010.docx]

Table S5. Candidate genes involved in alanine, aspartate and glutamate metabolism.

| **Gene** | **EC number** | **Number of unique transcripts** |
| --- | --- | --- |
| Alanine-glyoxylate transaminase | 2.6.1.44 | 2 |
| Aspartate carbamoyltransferase | 2.1.3.2 | 2 |
| Glutamine-fructose-6-phosphate transaminase (isomerizing) | 2.6.1.16 | 1 |
| Glutamine-pyruvate transaminase | 2.6.1.15 | 1 |
| Glanine transaminase | 2.6.1.2 | 1 |
| Aspartate transaminase | 2.6.1.1 | 4 |
| Carbamoyl-phosphate synthase (glutamine-hydrolyzing) | 6.3.5.5 | 2 |
| Asparagine synthase (glutamine-hydrolyzing) | 6.3.5.4 | 1 |
| Glutamate-ammonia ligase | 6.3.1.2 | 1 |
| Argininosuccinate synthase | 6.3.4.5 | 1 |
| Adenylosuccinate synthase | 6.3.4.4 | 1 |
| Alanine dehydrogenase | 1.4.1.1 | 2 |
