# Supplementary material for: Transcriptomic Study Reveals Widespread Spliced Leader Trans-Splicing, Short 5′-UTRs and Potential Complex Carbon Fixation Mechanisms in the Euglenoid Alga Eutreptiella sp
Source: PLoS One. 2013 Apr 9;8(4):e60826. doi: 10.1371/journal.pone.0060826 (PMC3621762; doi:10.1371/journal.pone.0060826)
Supplement: Table S6 — Candidate genes involved in fructose and mannose metabolism. (DOCX) [file pone.0060826.s011.docx]

Table S6. Candidate genes involved in fructose and mannose metabolism.

| **Gene** | **EC number** | **Number of unique transcripts** |
| --- | --- | --- |
| 6-phosphofructo-2-kinase | 2.7.1.105 | 1 |
| 6-phosphofructokinase | 2.7.1.11 | 1 |
| GDP-mannose 4,6-dehydratase | 4.2.1.47 | 5 |
| Fructose-2,6-bisphosphate 2-phosphatase | 3.1.3.46 | 1 |
| Phosphomannomutase | 5.4.2.8 | 1 |
| ADP-sugar diphosphatase | 3.6.1.21 | 1 |
| GDP-L-fucose synthase | 1.1.1.271 | 2 |
| Fructose-bisphosphatase | 3.1.3.11 | 2 |
| L-fucose isomerase | 5.3.1.25 | 1 |
| Fructose-bisphosphate aldolase | 4.1.2.13 | 5 |
| Aldehyde reductase | 1.1.1.21 | 3 |
| Mannose-6-phosphate isomerase | 5.3.1.8 | 1 |
| Triose-phosphate isomerase | 5.3.1.1 | 7 |
