# Supplementary material for: Transcriptomic Study Reveals Widespread Spliced Leader Trans-Splicing, Short 5′-UTRs and Potential Complex Carbon Fixation Mechanisms in the Euglenoid Alga Eutreptiella sp
Source: PLoS One. 2013 Apr 9;8(4):e60826. doi: 10.1371/journal.pone.0060826 (PMC3621762; doi:10.1371/journal.pone.0060826)
Supplement: Table S7 — Candidate genes involved in glycine, serine and threonine metabolism. (DOCX) [file pone.0060826.s012.docx]

Table S7. Candidate genes involved in glycine, serine and threonine metabolism.

| **Gene** | **EC number** | **Number of unique transcripts** |
| --- | --- | --- |
| Phosphoserine phosphatase | 3.1.3.3 | 1 |
| Glycine dehydrogenase (decarboxylating) | 1.4.4.2 | 1 |
| Serine-pyruvate transaminase | 2.6.1.51 | 1 |
| Serine-glyoxylate transaminase | 2.6.1.45 | 1 |
| Alanine-glyoxylate transaminase | 2.6.1.44 | 2 |
| Glycine hydroxymethyltransferase | 2.1.2.1 | 2 |
| L-serine ammonia-lyase | 4.3.1.17 | 3 |
| Cystathionine beta-synthase | 4.2.1.22 | 3 |
| Choline dehydrogenase | 1.1.99.1 | 1 |
| Threonine synthase | 4.2.3.1 | 1 |
| Dihydrolipoyl dehydrogenase | 1.8.1.4 | 1 |
| Phosphoglycerate mutase | 5.4.2.1 | 1 |
| Phosphoglycerate dehydrogenase | 1.1.1.95 | 5 |
| 5-aminolevulinate synthase | 2.3.1.37 | 1 |
| Primary-amine oxidase | 1.4.3.21 | 1 |
| Aspartate-semialdehyde dehydrogenase | 1.2.1.11 | 1 |
| L-threonine aldolase | 4.1.2.5 | 1 |
| Cystathionine gamma-lyase | 4.4.1.1 | 6 |
