# Supplementary material for: Transcriptomic Study Reveals Widespread Spliced Leader Trans-Splicing, Short 5′-UTRs and Potential Complex Carbon Fixation Mechanisms in the Euglenoid Alga Eutreptiella sp
Source: PLoS One. 2013 Apr 9;8(4):e60826. doi: 10.1371/journal.pone.0060826 (PMC3621762; doi:10.1371/journal.pone.0060826)
Supplement: Table S8 — Candidate genes involved in amino sugar and nucleotide sugar metabolism. (DOCX) [file pone.0060826.s013.docx]

Table S8. Candidate genes involved in amino sugar and nucleotide sugar metabolism.

| **Gene** | **EC number** | **Number of unique transcripts** |
| --- | --- | --- |
| Alpha-N-arabinofuranosidase | 3.2.1.55 | 1 |
| UTP-xylose-1-phosphate uridylyltransferase | 2.7.7.11 | 1 |
| UTP-hexose-1-phosphate uridylyltransferase | 2.7.7.10 | 1 |
| Xylan 1,4-beta-xylosidase | 3.2.1.37 | 1 |
| Glucokinase | 2.7.1.2 | 1 |
| Glutamine-fructose-6-phosphate transaminase (isomerizing) | 2.6.1.16 | 1 |
| UDP-glucose 4,6-dehydratase | 4.2.1.76 | 2 |
| GDP-mannose 4,6-dehydratase | 4.2.1.47 | 5 |
| N-acylglucosamine-6-phosphate 2-epimerase | 5.1.3.9 | 1 |
| UDP-glucose 4-epimerase | 5.1.3.2 | 1 |
| Phosphomannomutase | 5.4.2.8 | 1 |
| Phosphoglucomutase | 5.4.2.2 | 2 |
| UDP-glucuronate decarboxylase | 4.1.1.35 | 2 |
| GDP-L-fucose synthase | 1.1.1.271 | 2 |
| UTP-glucose-1-phosphate uridylyltransferase | 2.7.7.9 | 2 |
| UDP-glucose 6-dehydrogenase | 1.1.1.22 | 3 |
| Glucose-6-phosphate isomerase | 5.3.1.9 | 1 |
| Mannose-6-phosphate isomerase | 5.3.1.8 | 1 |
| Polyphosphate-glucose phosphotransferase | 2.7.1.63 | 1 |
| Glucuronate-1-phosphate uridylyltransferase | 2.7.7.44 | 1 |
| UDP-N-acetylmuramate dehydrogenase | 1.1.1.158 | 1 |
|  |  |  |
