# Supplementary material for: Transcriptomic Study Reveals Widespread Spliced Leader Trans-Splicing, Short 5′-UTRs and Potential Complex Carbon Fixation Mechanisms in the Euglenoid Alga Eutreptiella sp
Source: PLoS One. 2013 Apr 9;8(4):e60826. doi: 10.1371/journal.pone.0060826 (PMC3621762; doi:10.1371/journal.pone.0060826)
Supplement: Table S9 — Candidate genes involved in arginine and proline metabolism. (DOCX) [file pone.0060826.s014.docx]

Table S9. Candidate genes involved in arginine and proline metabolism.

| **Gene** | **EC number** | **Number of unique transcripts** |
| --- | --- | --- |
| Procollagen-proline dioxygenase | 1.14.11.2 | 4 |
| Ornithine carbamoyltransferase | 2.1.3.3 | 1 |
| Ornithine aminotransferase | 2.6.1.13 | 1 |
| Glutamate 5-kinase | 2.7.2.11 | 1 |
| Aspartate transaminase | 2.6.1.1 | 4 |
| Spermine synthase | 2.5.1.22 | 2 |
| Spermidine synthase | 2.5.1.16 | 3 |
| Glutamate-ammonia ligase | 6.3.1.2 | 1 |
| Diamine N-acetyltransferase | 2.3.1.57 | 1 |
| Glutamate-5-semialdehyde dehydrogenase | 1.2.1.41 | 1 |
| Argininosuccinate synthase | 6.3.4.5 | 1 |
| Proline dehydrogenase | 1.5.99.8 | 2 |
|  |  |  |
