# Supplementary material for: Transcriptomic Study Reveals Widespread Spliced Leader Trans-Splicing, Short 5′-UTRs and Potential Complex Carbon Fixation Mechanisms in the Euglenoid Alga Eutreptiella sp
Source: PLoS One. 2013 Apr 9;8(4):e60826. doi: 10.1371/journal.pone.0060826 (PMC3621762; doi:10.1371/journal.pone.0060826)
Supplement: Table S10 — Candidate genes involved in glutathione metabolism. (DOCX) [file pone.0060826.s015.docx]

Table S10. Candidate genes involved in glutathione metabolism.

| **Gene** | **EC number** | **Number of unique transcripts** |
| --- | --- | --- |
| Pyrimidodiazepine synthase | 1.5.4.1 | 1 |
| Peroxiredoxin | 1.11.1.15 | 7 |
| L-ascorbate peroxidase | 1.11.1.11 | 1 |
| Spermine synthase | 2.5.1.22 | 2 |
| Spermidine synthase | 2.5.1.16 | 3 |
| Ribonucleoside-diphosphate reductase | 1.17.4.1 | 1 |
| Glutathione dehydrogenase (ascorbate) | 1.8.5.1 | 1 |
| Glutathione-disulfide reductase | 1.8.1.7 | 1 |
| Glutathione peroxidase | 1.11.1.9 | 3 |
| Glucose-6-phosphate dehydrogenase | 1.1.1.49 | 1 |
| Phosphogluconate dehydrogenase (decarboxylating) | 1.1.1.44 | 3 |
| Isocitrate dehydrogenase (NADP+) | 1.1.1.42 | 3 |
| 5-oxoprolinase (ATP-hydrolysing) | 3.5.2.9 | 1 |
