# Supplementary material for: Transcriptomic Study Reveals Widespread Spliced Leader Trans-Splicing, Short 5′-UTRs and Potential Complex Carbon Fixation Mechanisms in the Euglenoid Alga Eutreptiella sp
Source: PLoS One. 2013 Apr 9;8(4):e60826. doi: 10.1371/journal.pone.0060826 (PMC3621762; doi:10.1371/journal.pone.0060826)
Supplement: Table S11 — Candidate genes involved in pyruvate metabolism. (DOCX) [file pone.0060826.s016.docx]

Table S11. Candidate genes involved in pyruvate metabolism.

| **Gene** | **EC number** | **Number of unique transcripts** |
| --- | --- | --- |
| Pyruvate, phosphate dikinase | 2.7.9.1 | 1 |
| Pyruvate dehydrogenase (acetyl-transferring) | 1.2.4.1 | 3 |
| Acetyl-CoA carboxylase | 6.4.1.2 | 2 |
| Formate C-acetyltransferase | 2.3.1.54 | 1 |
| Acetate-CoA ligase | 6.2.1.1 | 3 |
| Dihydrolipoyl dehydrogenase | 1.8.1.4 | 1 |
| Malate dehydrogenase (NADP+) | 1.1.1.82 | 1 |
| Dihydrolipoyllysine-residue acetyltransferase | 2.3.1.12 | 1 |
| Malate dehydrogenase | 1.1.1.37 | 6 |
| Aldehyde reductase | 1.1.1.21 | 3 |
| Lactoylglutathione lyase | 4.4.1.5 | 1 |
| Malate synthase | 2.3.3.9 | 1 |
| Pyruvate kinase | 2.7.1.40 | 1 |
| 2-isopropylmalate synthase | 2.3.3.13 | 5 |
