# Supplementary material for: Transcriptomic Study Reveals Widespread Spliced Leader Trans-Splicing, Short 5′-UTRs and Potential Complex Carbon Fixation Mechanisms in the Euglenoid Alga Eutreptiella sp
Source: PLoS One. 2013 Apr 9;8(4):e60826. doi: 10.1371/journal.pone.0060826 (PMC3621762; doi:10.1371/journal.pone.0060826)
Supplement: Table S12 — Candidate genes involved in purine metabolism. (DOCX) [file pone.0060826.s017.docx]

Table S12. Candidate genes involved in purine metabolism.

| **Gene** | **EC number** | **Number of unique transcripts** |
| --- | --- | --- |
| 5'-nucleotidase | 3.1.3.5 | 1 |
| Adenylyl-sulfate kinase | 2.7.1.25 | 1 |
| Adenosine kinase | 2.7.1.20 | 5 |
| Adenosine triphosphatase | 3.6.1.3 | 28 |
| Phosphoribosylaminoimidazolesuccinocarboxamide synthase | 6.3.2.6 | 2 |
| IMP cyclohydrolase | 3.5.4.10 | 2 |
| Phosphoribosylaminoimidazolecarboxamide formyltransferase | 2.1.2.3 | 2 |
| Ribonucleoside-diphosphate reductase | 1.17.4.1 | 1 |
| Nucleoside-diphosphate kinase | 2.7.4.6 | 4 |
| Adenylate kinase | 2.7.4.3 | 9 |
| Phosphoribosylformylglycinamidine synthase | 6.3.5.3 | 1 |
| Adenosine deaminase | 3.5.4.4 | 1 |
| Adenine deaminase | 3.5.4.2 | 1 |
| Phosphoglucomutase | 5.4.2.2 | 2 |
| ADP-sugar diphosphatase | 3.6.1.21 | 1 |
| Nucleoside-triphosphatase | 3.6.1.15 | 30 |
| GMP reductase | 1.7.1.7 | 2 |
| Phosphoribosylaminoimidazole carboxylase | 4.1.1.21 | 2 |
| DNA-directed DNA polymerase | 2.7.7.7 | 5 |
| DNA-directed RNA polymerase | 2.7.7.6 | 10 |
| Sulfate adenylyltransferase | 2.7.7.4 | 1 |
| Adenylosuccinate synthase | 6.3.4.4 | 1 |
| Guanylate cyclase | 4.6.1.2 | 1 |
| Adenylate cyclase | 4.6.1.1 | 10 |
| 3',5'-cyclic-nucleotide phosphodiesterase | 3.1.4.17 | 1 |
| IMP dehydrogenase | 1.1.1.205 | 2 |
| Ribose-phosphate diphosphokinase | 2.7.6.1 | 1 |
| Pyruvate kinase | 2.7.1.40 | 1 |
