# Supplementary material for: Transcriptomic Study Reveals Widespread Spliced Leader Trans-Splicing, Short 5′-UTRs and Potential Complex Carbon Fixation Mechanisms in the Euglenoid Alga Eutreptiella sp
Source: PLoS One. 2013 Apr 9;8(4):e60826. doi: 10.1371/journal.pone.0060826 (PMC3621762; doi:10.1371/journal.pone.0060826)
Supplement: Table S13 — Candidate genes involved in aminoacyl-tRNA biosynthesis. (DOCX) [file pone.0060826.s018.docx]

Table S13. Candidate genes involved in aminoacyl-tRNA biosynthesis.

| **Gene** | **EC number** | **Number of unique genes** |
| --- | --- | --- |
| Phenylalanine-tRNA ligase | 6.1.1.20 | 1 |
| Glutamine-tRNA ligase | 6.1.1.18 | 2 |
| Glutamate-tRNA ligase | 6.1.1.17 | 2 |
| Glycine-tRNA ligase | 6.1.1.14 | 1 |
| Serine-tRNA ligase | 6.1.1.11 | 2 |
| Lysine-tRNA ligase | 6.1.1.6 | 1 |
| Isoleucine-tRNA ligase | 6.1.1.5 | 1 |
| Tryptophan-tRNA ligase | 6.1.1.2 | 1 |
| Tyrosine-tRNA ligase | 6.1.1.1 | 1 |
