# Supplementary material for: Transcriptomic Study Reveals Widespread Spliced Leader Trans-Splicing, Short 5′-UTRs and Potential Complex Carbon Fixation Mechanisms in the Euglenoid Alga Eutreptiella sp
Source: PLoS One. 2013 Apr 9;8(4):e60826. doi: 10.1371/journal.pone.0060826 (PMC3621762; doi:10.1371/journal.pone.0060826)
Supplement: Table S14 — Candidate genes involved in pantothenate and CoA biosynthesis. (DOCX) [file pone.0060826.s019.docx]

Table S14. Candidate genes involved in pantothenate and CoA biosynthesis.

| **Gene** | **EC number** | **Number of unique transcripts** |
| --- | --- | --- |
| Dihydropyrimidinase | 3.5.2.2 | 1 |
| Dihydroxy-acid dehydratase | 4.2.1.9 | 8 |
| 3-methyl-2-oxobutanoate hydroxymethyltransferase | 2.1.2.11 | 2 |
| Branched-chain-amino-acid transaminase | 2.6.1.42 | 1 |
| Beta-ureidopropionase | 3.5.1.6 | 1 |
| Acetolactate synthase | 2.2.1.6 | 3 |
| Phosphopantothenoylcysteine decarboxylase | 4.1.1.36 | 3 |
| Ketol-acid reductoisomerase | 1.1.1.86 | 1 |
| Dihydropyrimidine dehydrogenase (NADP+) | 1.3.1.2 | 1 |
